# Characterization of the cyclic dipeptide cyclo(His-Pro) in Arabidopsis

**DOI:** 10.1093/plphys/kiaf174

**Published:** 2025-05-03

**Authors:** Romina I Minen, Maria Dolores Camalle, Tyler J Schwertfeger, Fatimah Abdulhakim, Hannah Reish, Leonardo Perez de Souza, Juan C Moreno, Anthony Schilmiller, Venkatesh P Thirumalaikumar, Pallavi Agarwal, Caroline F Plecki, Alisdair R Fernie, Heribert Hirt, Frank C Schroeder, Aleksandra Skirycz

**Affiliations:** Boyce Thompson Institute, Ithaca, NY 14853, USA; DKFZ German Cancer Research Center, Heidelberg 69120, Germany; Michigan State University, East Lansing, MI 48824, USA; Boyce Thompson Institute, Ithaca, NY 14853, USA; Cornell University, Ithaca, NY 14853, USA; Darwin21 Desert Research Initiative, Biological and Environmental Science and Engineering Division, King Abdullah University of Science and Technology (KAUST), Thuwal 23955-6900, Saudi Arabia; Michigan State University, East Lansing, MI 48824, USA; Max-Planck-Institute of Molecular Plant Physiology, Potsdam-Golm 14476, Germany; Darwin21 Desert Research Initiative, Biological and Environmental Science and Engineering Division, King Abdullah University of Science and Technology (KAUST), Thuwal 23955-6900, Saudi Arabia; Max-Planck-Institute of Molecular Plant Physiology, Potsdam-Golm 14476, Germany; Michigan State University, East Lansing, MI 48824, USA; Boyce Thompson Institute, Ithaca, NY 14853, USA; Purdue University, West Lafayette, IN 47907, USA; Boyce Thompson Institute, Ithaca, NY 14853, USA; Michigan State University, East Lansing, MI 48824, USA; Boyce Thompson Institute, Ithaca, NY 14853, USA; Syracuse University, Syracuse, NY 13244, USA; Max-Planck-Institute of Molecular Plant Physiology, Potsdam-Golm 14476, Germany; Darwin21 Desert Research Initiative, Biological and Environmental Science and Engineering Division, King Abdullah University of Science and Technology (KAUST), Thuwal 23955-6900, Saudi Arabia; Boyce Thompson Institute, Ithaca, NY 14853, USA; Cornell University, Ithaca, NY 14853, USA; Boyce Thompson Institute, Ithaca, NY 14853, USA; Michigan State University, East Lansing, MI 48824, USA

## Abstract

Diketopiperazines (DKPs) are chemically and functionally diverse cyclic dipeptides associated primarily with microbes. Few DKPs have been reported from plants and animals; the best characterized is cyclo(His-Pro), found in the mammalian central nervous system, where it arises from the proteolytic cleavage of a thyrotropin-releasing tripeptide hormone. Herein, we report the identification of cyclo(His-Pro) in Arabidopsis (*Arabidopsis thaliana*), where its levels increase upon abiotic stress conditions, including high salt, heat, and cold. To screen for potential protein targets, we used isothermal shift assays, which examine changes in protein-melting stability upon ligand binding. Among the identified proteins, we focused on the glycolytic enzyme, cytosolic glyceraldehyde-3-phosphate dehydrogenase (GAPC1). Binding between the GAPC1 protein and cyclo(His-Pro) was validated using nano-differential scanning fluorimetry and microscale thermophoresis, and we could further demonstrate that cyclo(His-Pro) inhibits GAPC1 activity with an IC_50_ of ∼200 *μ*m. This inhibition was conserved in human GAPDH. Inhibition of glyceraldehyde-3-phosphate dehydrogenase activity has previously been reported to reroute carbon from glycolysis toward the pentose phosphate pathway. Accordingly, cyclo(His-Pro) supplementation augmented NADPH levels, increasing the NADPH/NADP^+^ ratio. Phenotypic screening revealed that plants supplemented with cyclo(His-Pro) were more tolerant to high-salt stress, as manifested by higher biomass, which we show is dependent on GAPC1/2. Our work reports the identification and functional characterization of cyclo(His-Pro) as a modulator of glyceraldehyde-3-phosphate dehydrogenase in plants.

## Teaser

The cyclic dipeptide cyclo(His-Pro) binds to and inhibits the activity of the glycolytic enzyme glyceraldehyde-3-phosphate dehydrogenase in Arabidopsis, affecting plant stress tolerance.

## Introduction

Cyclization of dipeptides (Ortiz and Sansinenea 2017; Bushman et al. 2023; Jia et al. 2023) results in the formation of substituted diketopiperazines, which in bacteria and fungi are often modified further to produce a range of structurally and functionally diverse secondary metabolites. In bacteria, diketopiperazine (DKP) synthesis relies mainly on the activity of cyclic dipeptide synthetases (Gu et al. 2013; Skinnider et al. 2018), whereas fungi utilize nonribosomal protein synthetases (Gu et al. 2013). In bacteria, DKPs are discussed in the context of chemical communication, based on their anti-quorum sensing activity and their role as antibiotics (Bushman et al. 2023). Similarly, fungal DKPs were associated with mediating interactions with other organisms, and many have demonstrated antimicrobial, antifungal, and antiviral properties (Bushman et al. 2023). Microbiota-derived DKPs were shown to affect the health of both animals and plants. DKPs produced by gut microbiota play a role in human health as they influence microbiota composition and pathogen colonization (Ogilvie and Czekster 2023). Like animals, plants also host endophytic bacteria and fungi known to synthesize DKPs (Stelmasiewicz et al. 2023). In the best-described example, 3 proline-containing DKPs from the plant-associated bacteria, *Pseudomonas aeruginosa*, were shown to act as plant growth regulators. Arabidopsis supplementation with cyclo(Pro-Val), cyclo(Pro-Phe), and cyclo(Pro-Tyr) boosts root growth, with the most pronounced effects on the lateral root numbers. This phenotype was attributed to DKPs acting as auxin mimics (Ortiz-Castro et al. 2011), and also activation of the TARGET OF RAPAMYCIN (TOR) kinase signaling (González-López et al. 2021).

However, there are fewer examples of animal-derived DKPs, where they derive primarily from proteolytic breakdown followed by spontaneous cyclization of linear peptide precursors. The by far best characterized example is cyclo(His-Pro). First reported in the human central nervous system (CNS) (Prasad et al. 1977), cyclo(His-Pro) was later identified in various tissues and bloody fluids (Prasad 1988). In the CNS cyclo(His-Pro) originates from hypothalamic thyrotropin-releasing hormone (TRH), which is a tripeptide with an amino acid sequence of pyroglutamyl-histidyl-proline amide. The pyroglutamate residue is cleaved by a specific pyroglutamate aminopeptidase resulting in a dipeptide His-Pro, which undergoes cyclization to cyclo(His-Pro) (Matsui et al. 1979; Møss and Bundgaard 1990). Cyclo(His-Pro) can be transported across the plasma membrane, by members of the organic cation/carnitine transporters family, most notably OCT2 (Taubert et al. 2007). In animals, cyclo(His-Pro) was shown to exert antioxidant and anti-inflammatory effects by regulating the activity of the nuclear factor erythroid 2-related factor 2 (Nrf2). Nrf2 is a transcription factor central to cellular response against oxidative stress and is situated upstream of the transcription of a plethora of antioxidant genes (Minelli et al. 2012). Recent work proposes a model where cyclo(His-Pro) binding to chloride intracellular channel protein 1 modulates calcium signaling and consequently Nrf2 activity (Ko et al. 2023). Moreover, cyclo(His-Pro) suppresses proinflammatory action of the nuclear factor-κB (NF-κB) complex (Minelli et al. 2012). Nrf2 and NF-κB signaling are interlinked, and an imbalance in either of the pathways has been associated with diseases ranging from neurodegeneration to cancer (Ben-Neriah and Karin 2011). Due to its antioxidant, anti-inflammatory, and neuroprotective properties, combined with the ability to cross the blood–brain barrier, excellent cellular retention, and resistance against degradation by peptidases, cyclo(His-Pro) is considered a promising therapeutic lead.

The presence of cyclo(His-Pro) in higher animals and the reported growth-promoting activity of microbiota-derived proline-containing DKPs in plants motivated us to look for cyclo(His-Pro) in plant samples. The presence of cyclic dipeptides in processed plant products, such as soy sauce, has been previously reported and was attributed to cyclization of protein degradation–derived linear dipeptides during processing (van der Laan et al. 2021). Here, we demonstrate that cyclo(His-Pro) is present in the model plant Arabidopsis (*Arabidopsis thaliana*), where it regulates activity of the glycolytic enzyme, cytosolic glyceraldehyde-3-phosphate dehydrogenase (GAPC1). Cyclo(His-Pro) inhibits GAPC1 activity and affects NADPH levels and stress tolerance.

## Results

### Cyclo(His-Pro) is a plant metabolite and accumulates in response to stress

**Figure 1.**
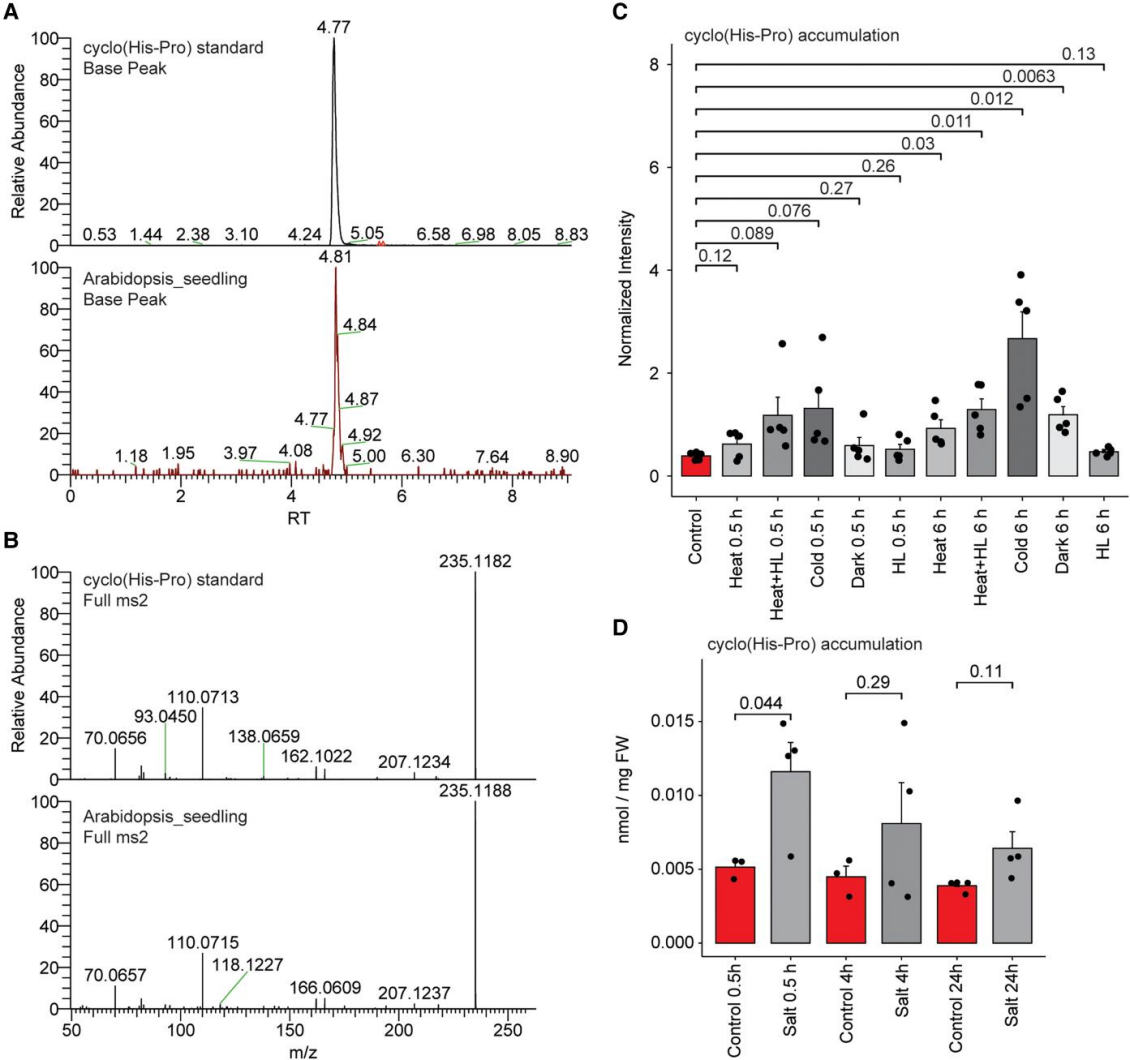
Cyclo(His-Pro) accumulates in response to stress in Arabidopsis. **A)** Detection of cyclo(His-Pro) via HILIC HPLC–HRMS. Cyclo(His-Pro) standard (upper panel) and Arabidopsis seedling sample (lower panel). Plants were grown on half-strength MS (0.5 MS) basal medium with 1% sucrose. **B)** Matching MS2 fragmentation pattern of the cyclo(His-Pro) standard (upper panel) and the corresponding *m*/*z* 235.12 metabolic feature measured in the Arabidopsis seedling sample (lower panel). **C)** Normalized intensity of cyclo(His-Pro) measured in the rosettes of 4- to 5-wk-old Arabidopsis plants subjected to different stress conditions at 0.5 and 6 h after stress onset. Data are mean ± Se of *n* = 5 where replicate is an independent plant from the same growth experiment. **D)** Absolute concentration (in nmol/mg FW) of cyclo(His-Pro) measured in the 12-d-old Arabidopsis seedlings grown on 0.5 MS medium transferred to 0.5 MS plates supplemented with 100 mm NaCl or without NaCl as a control. Samples were collected at 0.5, 4, and 24 h. Data are mean ± Se of *n* = 3 to 4, where replicate is an independent plate from the same growth experiment. **C** and **D)** Significance was estimated using unpaired 2-tailed Student's *t*-test. A significance threshold of 0.05 (or 5%) was used to determine whether a result is statistically significant. Graph was prepared using standard settings for a bar plot embedded in the SRplot web server (Tang et al. 2023).

To analyze metabolite extracts of Arabidopsis seedlings grown under axenic conditions for the presence of cyclo(His-Pro) and other DKPs, we used C_18_ HPLC–high-resolution mass spectrometry (HRMS; Alseekh et al. 2021) along with a series of authentic standards. Based on matching mass-to-charge ratios (*m*/*z*) and retention times (RTs) of standards and metabolic features detected in the Arabidopsis samples, we found a single hit, cyclo(His-Pro) (Supplementary Fig. S1A). As a means of independent validation, we also compared the cyclo(His-Pro) standard with Arabidopsis metabolite extract using orthogonal HILIC HPLC–HRMS method, and again we found a match based on RT, accurate mass, and fragmentation (Fig. 1, A and B). We also confirmed that the axenic media used in this experiment did not contain any cyclo(His-Pro), and thus, the cyclo(His-Pro) detected in the Arabidopsis samples must be of endogenous origin. Next, we queried a metabolomics dataset of 4- to 5-wk-old Arabidopsis subjected to 5 different stress conditions, harvested at 30 min and 6 h after stress onset, to investigate possible functions of cyclo(His-Pro) in Arabidopsis. We found that cyclo(His-Pro) concentrations were increased at the 6 h time point, especially in response to cold stress and, to a lesser extent, by a combination of heat and high light, heat stress alone, and darkness (Fig. 1C). Because the control samples for the stress experiment were taken exclusively at the stress onset, to exclude diel effects, we revisited an existing metabolomics dataset comprising data for 4- to 5-wk-old Arabidopsis rosettes harvested at 11 time points during the long-day diel cycle (Calderan-Rodrigues et al. 2021). Among the previously unknown metabolic features, we annotated cyclo(His-Pro), but measured no diel oscillations (Supplementary Fig. S1B). Finally, we estimated the cellular concentration of cyclo(His-Pro) in 12-d-old Arabidopsis seedlings grown on the 0.5 Murashige and Skoog (MS) medium transferred to either control or high-salt (100 mm NaCl) conditions for 0.5, 4, and 24 h (Fig. 1D). High-salt conditions increased cyclo(His-Pro) levels, with a highest concentration of ∼0.012 *μ*mol/g fresh weight (FW) measured at the 0.5 h time point. In comparison, the concentration of cyclo(His-Pro) in 4- to 5-wk-old Arabidopsis was significantly higher (*t*-test, *P* < 0.05); estimated to be ∼0.085 *μ*mol/g FW in plants grown under standard long-day conditions (Supplementary Fig. S1C). These results indicate that cyclo(His-Pro) as an endogenous plant metabolite is produced in response to diverse stress conditions.

### Identification of cyclo(His-Pro) protein targets

**Figure 2.**
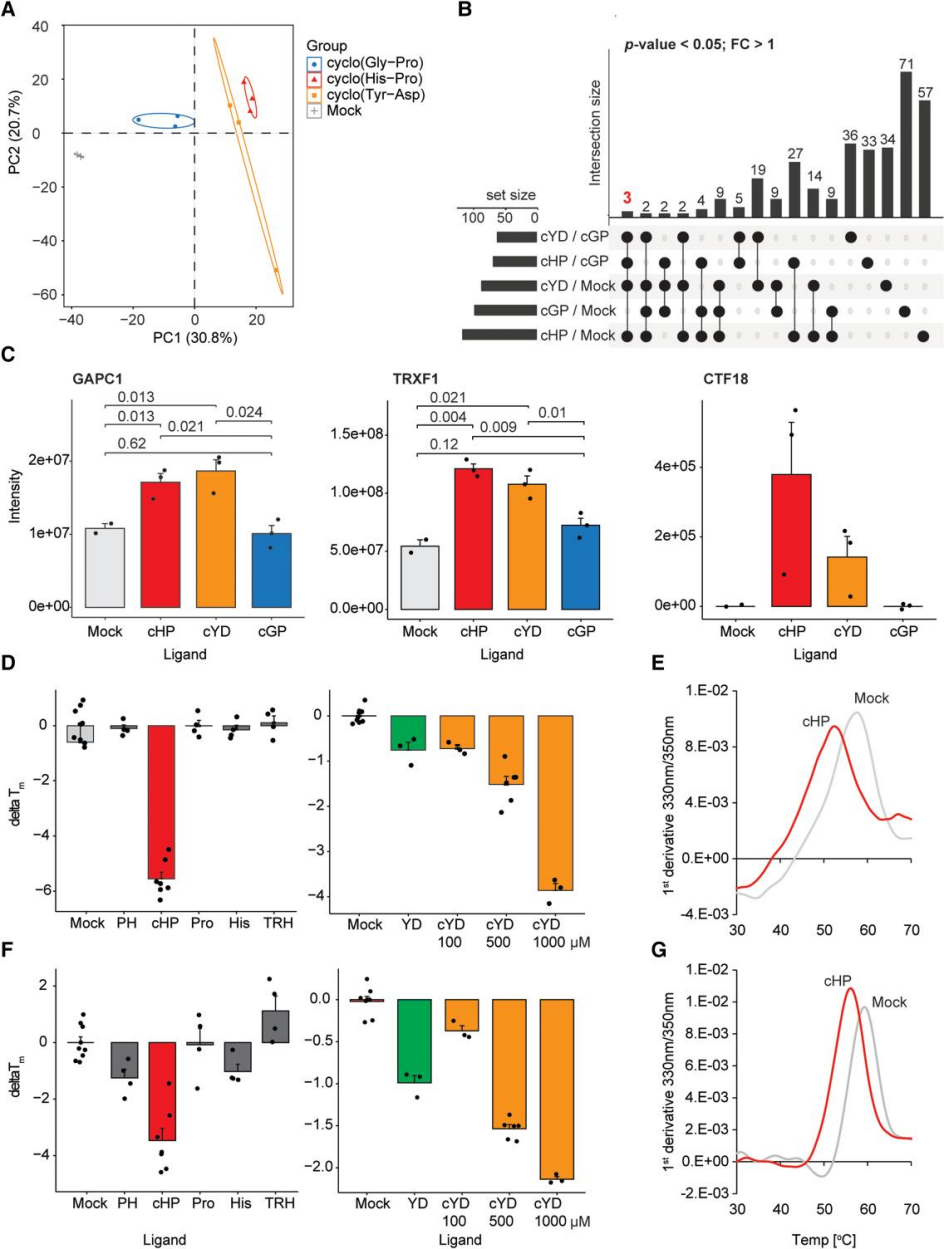
Cyclo(His-Pro) putative interactome comprises GAPC1. **A)** PCA was performed using all proteins present in the iTSA dataset, and the figure was prepared using standard settings for a PCA plot embedded in the SRplot web server (Tang et al. 2023). **B)** Upset plot representation of the differential proteins from the iTSA experiment (*t*-test, *P* < 0.05, FC > 1; *n* = 2 to 3). **C)** Protein intensities from the iTSA experiment. Data are mean ± Se of *n* = 2 to 3. **A** to **C)** Replicates represent independent plant lysates prepared from bulked seedlings from independent plant growth experiments. Significance was estimated using unpaired 2-tailed Student's *t*-test. Graphs were prepared using standard settings for a bar plot embedded in the SRplot web server (Tang et al. 2023). A significance threshold of 0.05 (or 5%) was used to determine whether a result is statistically significant. **D)** Difference in melting temperature of GAPC1 between mock samples and in the presence of the different ligands. The final concentration of the ligands was set to 100 *μ*m, with the exception of cyclo(Tyr-Asp) also measured at 500 *μ*m and 1 mm. Data are mean ± Se of *n* = 3 to 9, where replicates are independent capillaries. **E)** Representative melting profiles of the GAPC1 protein from a nanoDSF experiment. Here, for the protein alone (mock) and supplemented with 100 *μ*m cyclo(His-Pro). **F)** Difference in melting temperature of GAPDH between mock samples and in the presence of the different ligands. The final concentration of the ligands was set to 100 *μ*m, with the exception of cyclo(Tyr-Asp) also measured at 500 *μ*m and 1 mm. Data are mean ± Se of *n* = 3 to 9, where replicates are independent capillaries. **G)** Representative melting profiles of the GAPDH protein from a nanoDSF experiment. Here, for the protein alone (mock) and supplemented with 100 *μ*m cyclo(His-Pro). **C, D, F)** Graphs were prepared using standard settings for a bar plot embedded in the SRplot web server (Tang et al. 2023). cHP, cyclo(His-Pro); PH, Pro-His; Pro, proline; His, histidine; cYD, cyclo(Tyr-Asp); cGP, cyclo(Gly-Pro); YD, Tyr-Asp; TRH, thyrotropin-releasing hormone.

Metabolites exert many of their functions via binding to proteins (Kosmacz et al. 2020). Hence, to get further insight into cyclo(His-Pro) function, we employed the isothermal shift assay (iTSA), a proteomics MS method that detects shifts in the thermal stability of proteins induced by metabolite binding (Ball et al. 2020; Schlossarek et al. 2022). In short, the iTSA protocol involves heating a native lysate, with or without the studied ligand, to a single temperature selected based on the known median temperature of the proteome, followed by a short centrifugation. Denatured proteins migrate to the pellet, whereas proteins that are retained in the soluble fraction are measured using proteomics MS. Differential accumulation in the ligand versus control mock samples is interpreted as a shift in the thermal stability caused by the presence of a ligand. To investigate the specificity of any cyclo(His-Pro)-dependent changes in protein thermal stability, we included 2 cyclic dipeptides that were not detected in our plant samples as controls in the iTSA studies, cyclo(Gly-Pro) and cyclo(Tyr-Asp). We chose cyclo(Gly-Pro), because, similar to cyclo(His-Pro), it functions as a neuroactive peptide in eukaryotes (Guan and Gluckman 2009), and cyclo(Tyr-Asp) based on our previous work demonstrating the role of linear Tyr-Asp in regulating plant metabolism (Moreno et al. 2021). Principal component analysis (PCA) of the proteomics data showed a clear separation between plants treated with cyclo(His-Pro) and both mock and cyclo(Gly-Pro), but an intriguing similarity between cyclo(His-Pro) and cyclo(Tyr-Asp) treatments (Fig. 2A). Compared with the mock control, each dipeptide significantly affected thermal stability of several dozen proteins (*t*-test, *P* < 0.05; Fig. 2B, Supplementary Data Set 1). Among these, we prioritized proteins that contributed most significantly to the observed PCA separation. These analyses identified 3 proteins significantly stabilized by cyclo(His-Pro) as well as cyclo(Tyr-Asp), relative to mock control and cyclo(Gly-Pro) (Fig. 2C). In addition, 35 proteins were destabilized by cyclo(His-Pro) and cyclo(Tyr-Asp). The 3 stabilized proteins were a glycolytic enzyme, cytosolic glyceraldehyde-3-phosphate dehydrogenase (AT3G04120), known as GAPC in plants and GAPDH in animals (Plaxton 1996), type-f-thioredoxin (AT3G02730, TRFX-1), involved in the redox activation of carbon metabolism, including regulation of the plastidial glyceraldehyde-3-phosphate dehydrogenase (Buchanan and Balmer 2005), and a P-loop containing nucleoside triphosphate hydrolase (AT1G04730) required for sister chromatid cohesion (Fig. 2C). Destabilized proteins included several enzymes, ribosomal and proteasomal subunits, and actin-binding proteins. Finally, among the proteins stabilized exclusively by cyclo(His-Pro), we noted the presence of a putative pyroglutamyl peptidase (AT1G23440; Supplementary Data Set 1), which is of potential interest given the involvement of pyroglutamyl peptidases in cyclo(His-Pro) biogenesis in animals.

Among the identified proteins, we decided to follow-up on GAPC1, as regulation of glyceraldehyde-3-phosphate dehydrogenase activity was shown to play a central role in metabolic adaptation to oxidative stress (Ralser et al. 2007), which ties to the antioxidant properties of cyclo(His-Pro) reported in animals (Minelli et al. 2008). Moreover, we previously reported GAPC1 inhibition by a linear dipeptide, Tyr-Asp (Moreno et al. 2021), possibly related to the cyclo(Tyr-Asp) stabilization of the GAPC1 in iTSA experiments measured here. In order to independently test whether cyclo(His-Pro) and cyclo(Tyr-Asp) bind to GAPC1, the thermal stability of purified Arabidopsis GAPC1 (GAPC1) protein (Supplementary Fig. S2) was tested in the presence of 100 *μ*m cyclo(His-Pro), cyclo(Tyr-Asp), Pro-His, Tyr-Asp, proline, histidine, and TRH, using nano-differential scanning fluorimetry (nanoDSF). We did not include His-Pro in these assays because all available samples of the linear dipeptide contained a significant amount of cyclo(His-Pro), possibly formed by spontaneous cyclization. NanoDSF records a change in the fluorescence of tyrosine and tryptophan in the protein as it is subjected to steadily increasing temperatures. The temperature at which half of the protein is unfolded is referred to as the melting temperature (*T*_m_). Of the tested ligands, cyclo(His-Pro), cyclo(Tyr-Asp), and Tyr-Asp, but not Pro-His, TRH, or any of the single amino acids, significantly affected the GAPC1 melting temperature (Fig. 2, D and E). The *T*_m_ shift was also noticeably higher for cyclo(His-Pro) in comparison with cyclo(Tyr-Asp) and Tyr-Asp. The >4 °C difference measured for 100 *μ*m cyclo(His-Pro) could only be recapitulated at 1 mm cyclo(Tyr-Asp). As cyclo(His-Pro) is also present in animals, we purified human GAPDH to test for evolutionary conservation of glyceraldehyde-3-phosphate dehydrogenase inhibition. Note that GAPC and GAPDH are abbreviations of glyceraldehyde-3-phosphate dehydrogenase; GAPC used in plants and GAPDH in animals. Similar to GAPC1, cyclo(His-Pro), cyclo(Tyr-Asp), and Tyr-Asp significantly affected the GAPDH melting temperature (Fig. 2, F and G). These results point to direct binding of these ligands to GAPC1 and GAPDH.

### Cyclo(His-Pro) inhibits GAPC1 activity

**Figure 3.**
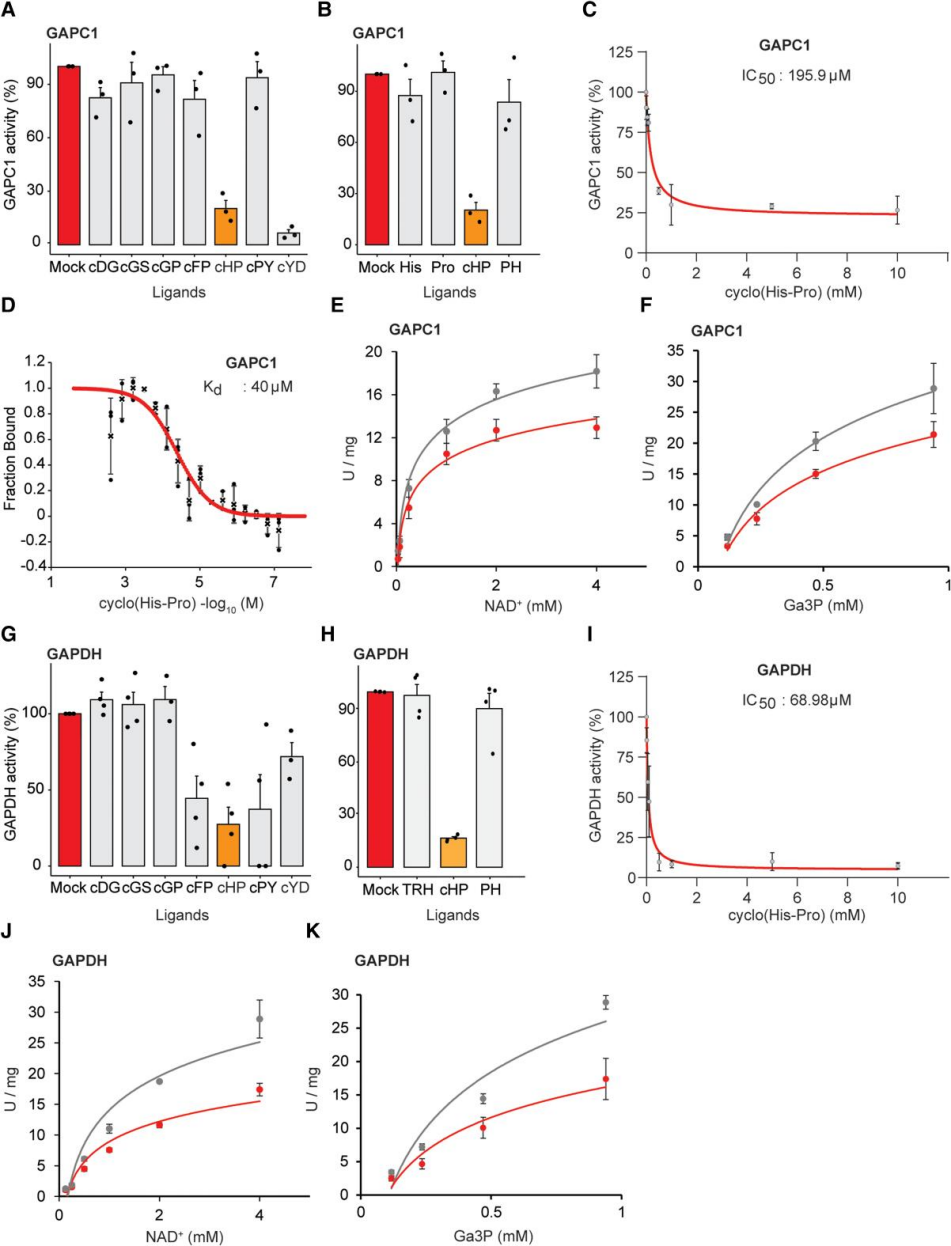
Effect of cyclic dipeptides on GAPC1 activity. **A** to **C)** GAPC1 enzymatic activity in the presence of cyclic and linear dipeptides and amino acids. **D)** Cyclo(His-Pro) binding to GAPC1 determined using microscale thermophoresis. Binding was derived from the spectral shift A670/A650 and expressed as fraction bound. **E** and **F)** GAPC1 enzymatic activity was measured in the varying concentrations of substrates in the absence (gray) and presence of 0.5 mm cyclo(His-Pro) (red). **G** to **I)** GAPDH enzymatic activity in the presence of cyclic and linear dipeptides and amino acids. **J** and **K)** GAPDH enzymatic activity was measured in the varying concentrations of substrates in the absence (gray) and presence of 0.5 mm cyclo(His-Pro) (red). **A, B, G,** and **H)** Ligands were tested at 10 mm final concentration, except for cyclo(Pro-Tyr) and cyclo(Tyr-Asp) at 5 mm. **C** and **I)** IC_50_ determined for cyclo(His-Pro) inhibition of GAPC1 and GAPDH enzymatic activity. **A** to **K)** Data are mean ± Se of *n* = 3 to 4, where replicates come from independent kinematic measurements or titrations (MST). **A, B, G,** and **H)** Graphs were prepared using standard settings for a bar plot embedded in the SRplot web server (Tang et al. 2023). **C** and **I)** Graphs were prepared using standard settings in GraphPad Prism, including IC_50_ curve fitting. **D)** Binding curve and *K_d_* were estimated using MonolithX (MST) analysis software. **E, F, J,** and **K)** Graphs were prepared in Excel. cHP, cyclo(His-Pro); PH, Pro-His; Pro, proline; His, histidine; cYD, cyclo(Tyr-Asp), cGP, cyclo(Gly-Pro); cDG, cyclo(Asp-Gly); cGS, cyclo(Gly-Ser); cFP, cyclo(Phe-Pro); cPY, cyclo(Pro-Tyr); Ga3P, glyceraldehyde-3-phosphate; NAD^+^, nicotinamide adenine dinucleotide.

To characterize the functional consequences of cyclo(His-Pro) binding, we tested whether cyclo(His-Pro) affects the enzymatic activity of purified GAPC1. For this, we monitored the oxidation of glyceraldehyde-3-phosphate and the reduction of NAD^+^ to NADH. Cyclo(His-Pro), but not Pro-His, histidine, and proline, inhibited GAPC1 activity (Fig. 3, A to C). The IC_50_ of the cyclo(His-Pro) was estimated to be ∼200 *μ*m. The IC_50_ agrees with the cyclo(His-Pro)-GAPC1-binding affinity determined by microscale thermophoresis (MST) of ∼40 *μ*m (Fig. 3D). Moreover, cyclo(His-Pro) inhibition of GAPC1 activity was independent of the NAD^+^ or Ga3P concentrations, suggesting noncompetitive binding (Fig. 3, E and F). Analogously to GAPC1, GAPDH is inhibited by cyclo(His-Pro) with an IC_50_ of 70 *μ*m, and the inhibition is independent of NAD^+^ or Ga3P concentrations (Fig. 3, G to K). We also tested the cyclo(His-Pro) precursor, TRH, but detected no change in GAPDH activity. The only other cyclic dipeptide that inhibited both GAPC1 and GAPDH was cyclo(Tyr-Asp), but the estimated IC_50_ of 1.5 mm is nearly 10-fold higher, indicating much weaker binding (Supplementary Fig. S3A). Finally, we found that GAPC1 is not inhibited by any of the other tested amino acids (Supplementary Fig. S3B). Among the 27 tested linear dipeptides, reproducible inhibition was observed for Gln-Pro and Tyr-Asp (Supplementary Fig. S3C). These results show that a specific set of linear and cyclic dipeptides, but not amino acids, inhibit GAPC1 activity, among which cyclo(His-Pro) is the most potent, representing an evolutionarily conserved glyceraldehyde-3-phosphate dehydrogenase inhibitor.

### Cyclo(His-Pro) treatment increases the NADPH/NADP^+^ ratio

**Figure 4.**
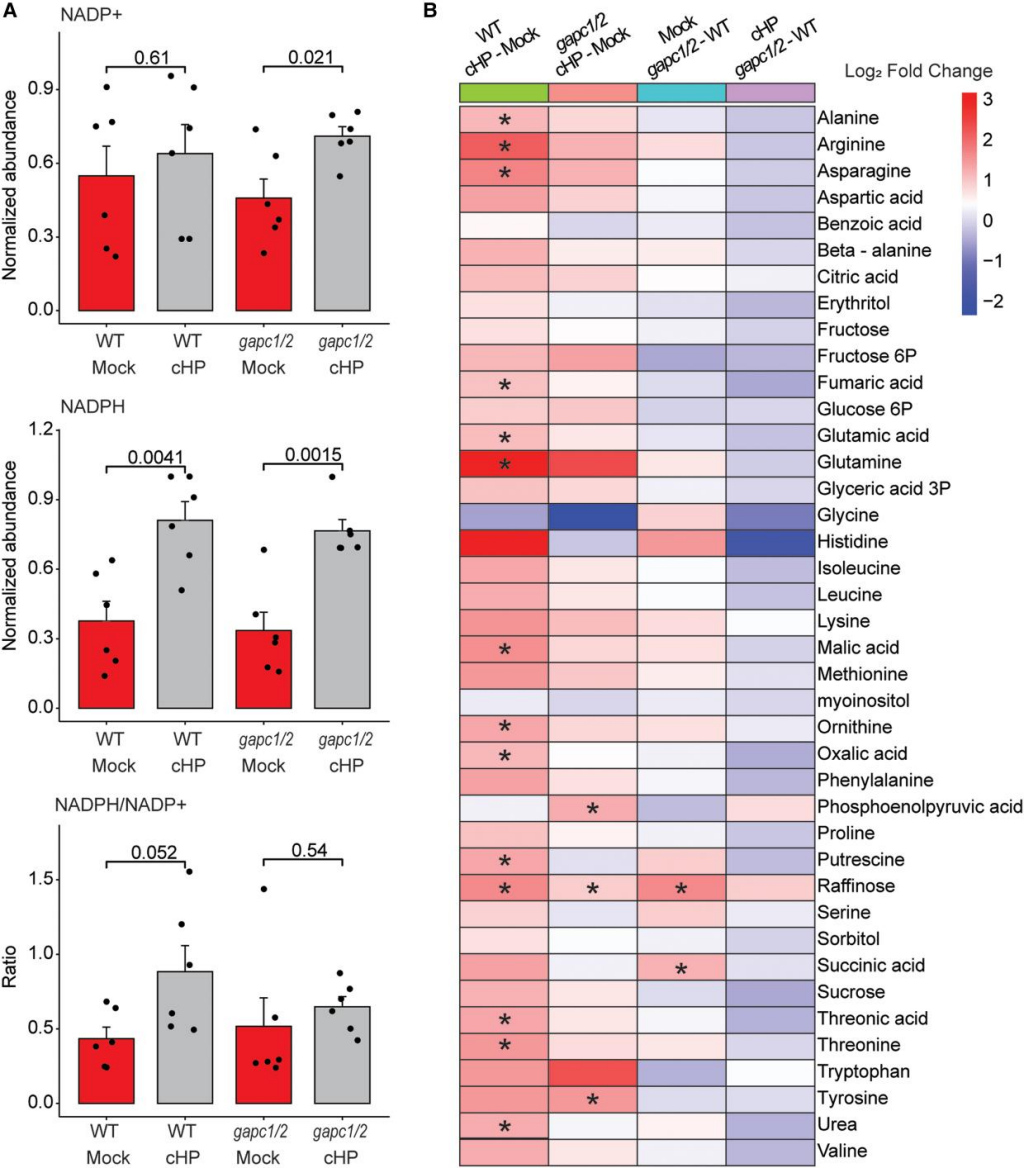
Cyclo(His-Pro) supplementation increases NADPH/NADP^+^ ratio and amino acids and polyamine levels. **A)** NADP^+^ and NADPH levels measured using enzyme cycling−based colorimetric assays were used to calculate NADPH/NADP^+^ ratios. Data are mean ± Se of *n* = 6, whereby replicates represent seedlings grown and treated in independent 6-well plates from 2 growth experiments. NADP^+^ and NADPH abundance was normalized to the maximum abundance measured across all samples in a growth experiment. Graphs were prepared using standard settings for a bar plot and heat map embedded in the SRplot web server (Tang et al. 2023). **B)** Heat map representing log_2_ fold-change accumulation of metabolites measured by GC–MS in the cyclo(His-Pro) supplementation experiment. Asterisks indicate significance (*t*-test, *P* < 0.05). Data are mean of *n* = 4, where replicates come from seedlings grown and treated in independent 6-well plates in a single growth experiment. **A** and **B)** Significance was estimated using unpaired 2-tailed Student's *t*-test. A significance threshold of 0.05 (or 5%) was used to determine whether a result is statistically significant. WT, wild-type plants; cHP, cyclo(His-Pro); NADP, nicotinamide adenine dinucleotide phosphate.

We and others have previously demonstrated that inhibition of GAPDH activity can redirect the flux from glycolysis to the pentose phosphate pathway (PPP) and, consequently, lead to an increased NADPH/NADP^+^ ratio (Ralser et al. 2007; Moreno et al. 2021; Talwar et al. 2023). Hence, we hypothesized that the cyclo(His-Pro) inhibition of GAPC1 reported here would produce a similar metabolic phenotype. To test our hypothesis, we supplemented Arabidopsis seedlings with 100 *μ*m cyclo(His-Pro) and harvested plant tissue to measure NADP^+^ and NADPH levels. Specifically, we harvested the plants at 4 h after cyclo(His-Pro) supplementation, and the entire experiment was performed in darkness, as photosynthesis is the primary source of reducing equivalents, rather than PPP, during the day. To test whether any supplementation phenotype depends on the cyclo(His-Pro) inhibition of GAPC1, we introduced a *gapc1gapc2* (*gapc1/2*) loss-of-function knockout mutant in 2 cytosolic GAPC isoenzymes, GAPC1 and GAPC2 (Guo et al. 2012). The *gapc1/2* mutant has been extensively characterized, which, among other phenotypes, revealed an increased NADPH/NADP^+^ ratio in developing seeds (Guo et al. 2014). As expected, cyclo(His-Pro) supplementation of wild-type plants resulted in an increase in the NADPH/NADP^+^ ratio, driven by significantly increased levels of NADPH (*t*-test, *P* < 0.05; Fig. 4A). In contrast to previous studies, levels of NADPH, NADP^+^, and the NADPH/NADP^+^ ratio did not differ between wild-type and *gapc1/2* plants in our experiments, which could be due to different conditions. Cyclo(His-Pro) treatment increased NADPH levels in the *gapc1/2* mutant to the same extent as in wild type, but the NADPH/NADP^+^ ratio was lower in cyclo(His-Pro)-treated *gapc1/2* mutants than in cyclo(His-Pro)-treated wild-type plants, indicating that inhibition of GAPC1 activity contributes to the observed increased NADPH/NADP^+^ ratio in the cyclo(His-Pro)-treated plants. Because of their role in regenerating NADPH and regulating carbon flux through the PPP, we also measured the effect of cyclo(His-Pro) supplementation on the activity of recombinant Arabidopsis glucose 6-phosphate dehydrogenase and 6-phosphogluconate dehydrogenase. However, in vitro and at concentrations of up to 10 mm of cyclo(His-Pro), we observed no effect on the activity of either enzyme.

To complement the NADPH and NADP^+^ measurements, we next profiled levels of primary metabolites using gas chromatography–MS (GC/MS; Fig. 4B, Supplementary Data Set 2). Many of the metabolites we found to be significantly increased upon cyclo(His-Pro) treatment represent nitrogen-rich amino acids and their derivatives, including arginine, asparagine, glutamic acid, glutamine and ornithine, the polyamine putrescine, and the arginine degradation product urea. Moreover, *gapc1/2* also responded to the cyclo(His-Pro), but similar to what we had found when measuring the NADPH/NADP^+^ ratio, the magnitude of change was smaller and not significant. In summary, cyclo(His-Pro) supplementation increases the NADPH/NADP^+^ ratio and levels of nitrogen-rich metabolites. Although GAPC1 inhibition contributes to these effects, it appears unlikely to be the sole driver of the observed metabolic phenotype.

### Cyclo(His-Pro) treatment improves stress tolerance

**Figure 5.**
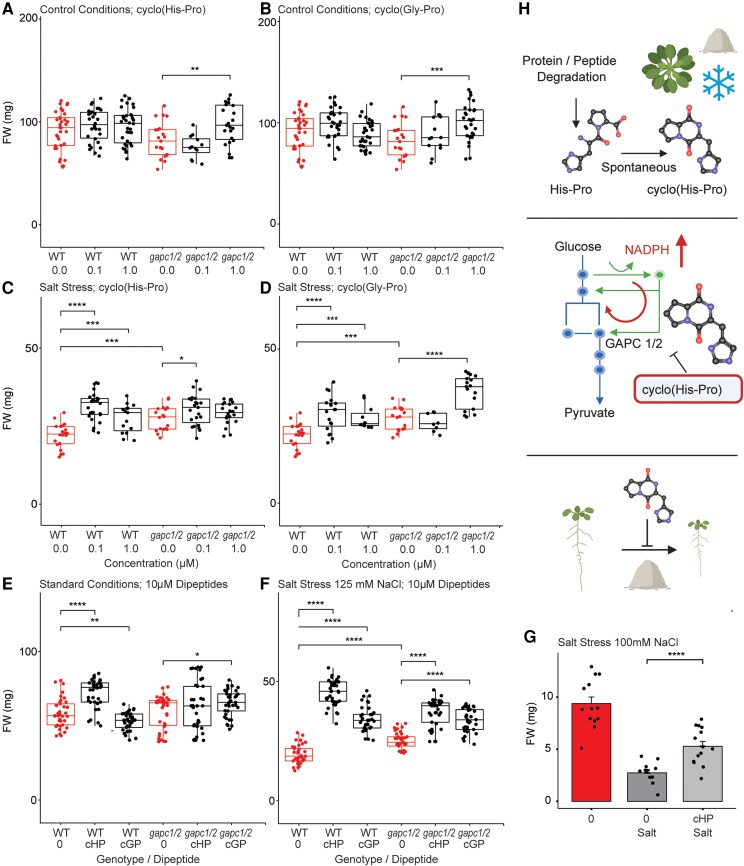
Cyclo(His-Pro) supplementation improves salt-stress tolerance. FW of Arabidopsis plants grown under standard **(A** and **B)** and high-salt conditions **(C** and **D)** without and with cyclo(His-Pro) and cyclo(Gly-Pro) at 0.1 and 1 *μ*m. Data are from *n* = 8 to 37, where replicate is an independent plant from the same growth experiment. **E** and **F)** FW of Arabidopsis plants grown under standard **(E)** and high-salt conditions **(F)** without and with cyclo(His-Pro) and cyclo(Gly-Pro) at 10 *μ*m. Data are from *n* = 37, where replicate is an independent plant from the same growth experiment. Measurements were taken from 3-wk-old plants transferred to either control or high-salt plates at 5 d old. **G)** FW of Arabidopsis seedlings grown under standard and high-salt conditions without and with cyclo(His-Pro) at 100 *μ*m. Data are mean ± Se of *n* = 11 to 14, where replicate is an independent plant from the same growth experiment. Measurements were taken from 12-d-old seedlings. **A** to **G)** Significance was estimated using unpaired 2-tailed Student's *t*-test; *P < 0.05, **P < 0.01, ***P < 0.001, ****P < 0.0001. Figures were prepared using standard settings for a box plot **(E** and **F)** and bar plot **(G)** embedded in the SRplot web server (Tang et al. 2023). For box plot: within each box, horizontal lines denote median values; boxes extend from the 25th to the 75th percentile of each group's distribution of values; vertical extending lines denote the most extreme values within 1.5 interquartile range; points correspond to individual measurements. **H)** Schematic representation of cyclo-His-Pro metabolism and mode of action. In Arabidopsis, cyclo(His-Pro) accumulates in response to cold and salt stress. Based on what is known in animals, we speculate that cyclo(His-Pro) arises from spontaneous cyclization of a linear dipeptide His-Pro, which is a product of protein degradation (upper panel). cyclo(His-Pro) binding inhibits the activity of glyceraldehyde-3-phosphate dehydrogenase redirecting glycolytic flux to PPP and NADPH production. Blue arrows indicate glycolysis and green PPP. Red arrow indicates a redirection of the flux upon GAPC inhibition (middle panel). Cyclo(His-Pro) supplementation mitigates growth penalty associated with salt stress (lower panel). Pointed arrows indicate activation, increase, or directionality, whereas flat arrows indicate inhibition. Figure was prepared using BioRender. WT, wild-type plants; cHP, cyclo(His-Pro); cGP, cyclo(Gly-Pro); FW, fresh weight; NADPH, nicotinamide adenine dinucleotide phosphate (reduced).

NADPH is a critical reducing equivalent, and increases in the NADPH/NADP^+^ ratio have been shown to be associated with improved stress tolerance in plants and animals (Ralser et al. 2007; Moreno et al. 2021). Therefore, we speculated that cyclo(His-Pro) supplementation may also improve stress tolerance in plants. To test this, we transferred 5-d-old Arabidopsis seedlings to either control or high-salt media, with and without cyclo(His-Pro), and measured the plant's FW after 16 d of treatment as a measure of fitness. As above, we tested the *gapc1/2* mutant and wild type. In addition to cyclo(His-Pro), we also tested cyclo(Gly-Pro), which did not inhibit GAPC1 activity. Under control conditions, we observed no significant effect of cyclo(His-Pro) or cyclo(Gly-Pro) on the wild-type FW (*t*-test, *P* < 0.05; Fig. 5, A and B). Salt dramatically reduced plant growth; however, the growth penalty was reduced in wild-type plants supplemented with cyclo(His-Pro) and cyclo(Gly-Pro) at 100 nm and 1 *μ*m concentrations (Fig. 5, C and D). Compared with the wild type, the *gapc1/2* mutant was less strongly affected by salt stress. The results are in line with previous work that showed that *gapc1/2* plants grow better under drought (Guo et al. 2012) and oxidative stress conditions (Moreno et al. 2021). Importantly, in addition to its role as a glycolytic enzyme, GAPC1 is known to have moonlighting roles relevant to plant response to stress. For instance, by interacting with phospholipase Dδ, GAPC1 has been linked to transduction of hydrogen peroxide signals (Guo et al. 2012). More recently, GAPC1 was also shown to act as a transcriptional regulator (Kim et al. 2020). Hence, the improved stress tolerance of *gapc1/2* plants may stem from more than alterations in redox metabolism. Significantly for our question, cyclo(His-Pro) supplementation of the *gapc1/2* mutant produced no further improvement of salt tolerance. In comparison, 1 *μ*m concentration of cyclo(Gly-Pro) did further increase *gapc1/2* salt tolerance. In a follow-up experiment, we increased cyclo(His-Pro) and cyclo(Gly-Pro) concentrations to 10 *μ*m (Fig. 5, E and F). In control conditions, cyclo(His-Pro), but not cyclo(Gly-Pro), increased the FW of the wild-type plants. Under salt conditions, both cyclo(His-Pro) and cyclo(Gly-Pro) alleviated the growth penalty associated with stress, resulting in bigger plants. This beneficial effect on plant growth was especially pronounced for cyclo(His-Pro). As in the first experiment, *gapc1/2* mutant was less strongly affected by salt stress than the wild type. Supplementation with cyclo(His-Pro) and cyclo(Gly-Pro) reduced the growth penalty associated with the salt stress, however, to a lesser extent than in the wild-type plants. Finally, we tested the effect of cyclo(His-Pro) on plants germinated and grown on high salt, and again, we found that cyclo(His-Pro) improved Arabidopsis salt-stress tolerance (Fig. 5G). Taken together, our results support a role of cyclo(His-Pro) inhibition of GAPC1 activity for the increased salt-stress tolerance associated with cyclo(His-Pro) supplementation, although we cannot exclude that at higher cyclo(His-Pro) concentrations additional targets may contribute. Notably, high concentrations of cyclo(Gly-Pro) also mitigate the growth penalty caused by salt stress, consistent with previously reported health-promoting effects of cyclic dipeptides on plant growth and stress resilience (Ortiz-Castro et al. 2011; Noh et al. 2017; Hung et al. 2024).

## Discussion

Cyclic dipeptides, a diverse group of compounds primarily produced by microbes, display a wide range of bioactivities in plants and animals. In plants, the best characterized are the proline-containing DKPs, cyclo(Pro-Val), cyclo(Pro-Phe), and cyclo(Pro-Tyr) from the plant bacteria *P. aeruginosa* PAO1, which stimulate root growth, particularly that of lateral roots (Ortiz-Castro et al. 2011). This effect was attributed to increased cell proliferation and found to be related to auxin and TOR signaling. In Arabidopsis and maize, these DKPs were shown to activate TOR, manifested by the phosphorylation of the S6 kinase, which participates in growth control (Corona-Sánchez et al. 2019; González-López et al. 2021). However, the exact mechanism by which TOR is activated remains to be studied. A possible mechanism may involve auxin: cyclo(Pro-Val), cyclo(Pro-Phe), and cyclo(Pro-Tyr) have been proposed to act as auxin mimics by directly binding to the auxin receptor TIR1. Auxin is known to activate TOR (Schepetilnikov et al. 2017), and moreover, auxin and TOR signaling interplay is crucial in lateral root development (Stitz et al. 2023). Meanwhile a different cyclic dipeptide cyclo(Ala-Gly) produced by the endophytic strain *Priestia megaterium* BP01R2, isolated from the Taiwanese salt marsh plant, *Bolboschoenus planiculmis*, alleviates the root growth penalty associated with salt stress (Hung et al. 2024), whereas cyclo(Gly-Pro), cyclo(Ala-Ile), cyclo(Ala-Leu), and cyclo(Leu-Pro) induce resistance against *Pseudomonas syringae* infection in Arabidopsis (Noh et al. 2017). Through these examples, DKPs emerge as an important group of small molecule compounds promoting plant growth and stress resilience. In contrast to the above work focusing on microbe-derived DKPs, we decided to study cyclo(His-Pro), one of the few cyclic dipeptides known to be produced by higher organisms, which we could also identify in Arabidopsis. We found that analogous to what has been reported in animals, cyclo(His-Pro) has beneficial effects on organismal fitness by mitigating damage related to stress.

Specifically, we focused our functional analysis on one of the cyclo(His-Pro) targets, GAPC1. Rapid and reversible regulation of glyceraldehyde-3-phosphate dehydrogenase activity is critical under oxidative stress conditions. Specifically, glyceraldehyde-3-phosphate dehydrogenase inactivation favors carbon flow toward PPP and NADPH regeneration (Ralser et al. 2007, 2009). NADPH boost is a key component of the organismal response to oxidative stress (Dick and Ralser 2015). NADPH is a cofactor of the enzymes involved in ROS scavenging and is required to replenish the pool of reduced glutathione (Ying 2008). The rapid inhibition of glyceraldehyde-3-phosphate dehydrogenase activity in response to stress is the result of reversible oxidation of catalytic cysteine residues. Persistent glutathionylation, however, was shown to destabilize GAPC1 conformation, promoting the formation of insoluble aggregates (Zaffagnini et al. 2019). Previously, we reported that in addition to oxidation, GAPC1 activity can also be inhibited by the linear dipeptide Tyr-Asp (Moreno et al. 2021). Plants treated with Tyr-Asp were characterized by a change in carbon flux from glycolysis toward PPP, increase in the NADPH/NADP^+^ ratio, and improved tolerance toward oxidative and salt stress. Similar to cyclo(His-Pro), which we showed here accumulates in response to stress (Fig. 5H), the levels of Asp and Glu-containing dipeptides are also increased in plants subjected to unfavorable environmental conditions, such as heat and darkness (Thirumalaikumar et al. 2020; Moreno et al. 2021). We proposed that dipeptide regulation may constitute an additional mechanism to inhibit glyceraldehyde-3-phosphate dehydrogenase activity, especially under prolonged stress conditions, to mitigate damage related to protein aggregation caused by persistent oxidation. Here, we demonstrate that Tyr-Asp is not the sole dipeptide derivative that regulates GAPC1 activity and, when supplemented, affects NADPH production and improves stress tolerance (Fig. 5H). Among the investigated linear and cyclic dipeptides, and based on the in vitro characterization of GAPC1 enzymatic activity, cyclo(His-Pro) is the most potent inhibitor with binding affinity and IC_50_ estimated at around 40 and 200 *μ*m, respectively, which is also in line with the estimated cellular concentrations. It has to be noted, however, that the binding affinity and IC_50_ are derived from in vitro characterization and may differ significantly in the cell. Moreover, our estimation of cellular concentration does not consider that concentrations likely vary in a cell-specific or cell-compartment-specific manner. These results beg the question to what extent physiological concentrations of cyclo(His-Pro) and the other dipeptide derivatives contribute to glyceraldehyde-3-phosphate dehydrogenase inhibition and NADPH production in vivo, e.g. under stress conditions. Identifying the dipeptide-binding sites in GAPC1 could help address questions about specificity and open a way for genetic manipulation to probe physiological relevance, in addition to cell type or cell-compartment-specific metabolomics to determine whether GAPC1 and cyclo(His-Pro) co-localize.

Significantly, supplementation with as low as 100 nm cyclo(His-Pro) concentrations mitigated the salt-stress-associated growth penalty, compared with the micromolar concentrations necessary for Tyr-Asp (Moreno et al. 2021). This is a significant result, and in the future, it would be interesting to test the effectiveness of cyclo(His-Pro) supplementation across a wider range of stress conditions. Given its excellent characteristics, such as ease of uptake and resistance to degradation, cyclo(His-Pro) constitutes an attractive compound to study in the context of plant stress resilience. In animals, cyclo(His-Pro) is predominantly formed from TRH (Minelli et al. 2008). TRH is a tripeptide composed of pyroglutamic acid, histidine, and proline. Pyroglutamic acid is cleaved by pyroglutamyl aminopeptidases, followed by nonenzymatic cyclization of the dipeptide His-Pro (Fig. 5H). His-Pro can also form during protein degradation (Fig. 5H). The source of cyclo(His-Pro) in plants is unknown, but interestingly, we found pyroglutamyl aminopeptidase among proteins affected by cyclo(His-Pro) in the iTSA experiment. Identifying proteins involved in cyclo(His-Pro) metabolism is an important future direction for understanding the function and specificity of cyclo(His-Pro) accumulation.

## Materials and methods

### Reagents

Cyclo(L-histidyl-L-proline) and cyclo(L-glycyl-L-proline) used in this study were purchased from Sigma-Aldrich. Linear L-dipeptides and L-amino acids were either purchased from Sigma-Aldrich or custom synthesized by GenScript. Synthesis of cyclo(l*-*tyrosyl*-*l*-*aspartic acid) is detailed in Supplementary Information (SI).

### Plant material

Seeds of the *gapc1gapc2* double mutant were kindly provided by Prof. Sam Wang from the Donald Danforth Center.

### Metabolite extraction for LC/MS and GC/MS analyses

Frozen plant samples were pulverized using a ball mill (Retsch, Germany). Approximately 25 mg of pulverized sample was aliquoted to each tube. One milliliter of precooled (−20 °C) extraction solution (methyl *tert*-butyl ether/methanol/water at 3:1:1) was added to the sample, followed by vortexing and 5 to 10 min sonication in a water sonication bath. Next, 0.5 mL of a precooled (4 °C) 3:1 water/methanol mixture was added to the tube and mixed by inversion. Samples were centrifuged for 5 min at 20,817 × *g* at room temperature. This yielded 2 separate liquid phases and a protein pellet. The upper phase and interphase were removed by vacuum aspiration. The remaining lower polar phase was transferred to a new tube and dried using a centrifugal evaporator. Protein pellets were washed with 0.5 mL methanol and left to dry at room temperature. Dried metabolite and protein samples were stored in −80 °C.

### C_18_ HPLC–HRMS

Samples from the stress experiment were measured, as described in Giavalisco et al. (2011), using ultra HPLC coupled to a Q-Exactive mass spectrometer (Thermo Fisher Scientific) in positive ionization mode. Metabolic features and the relative intensities were extracted from the chromatograms using Genedata software, as described in Schlossarek et al. (2022). Cyclo(His-Pro) was identified using *m*/*z* and RT information.

### HILIC UPLC–MS/MS

UPLC-MS/MS was performed using a Thermo Q-Exactive mass spectrometer and a Thermo Vanquish UHPLC. Five microliters of sample were injected onto a Waters Acquity Premier BEH-Amide column (2.1 × 100 mm) held at 40 °C. Compounds were separated using the following gradient run at 0.3 mL/min: initial conditions were 98% mobile Phase B (95% acetonitrile/5% water + 10 mm ammonium formate) and 2% mobile Phase A (10 mm ammonium formate in water), hold for 1 min at 2% A then ramp to 60% A at 6 min, hold at 60% A until 7 min, return to 2% A at 7.01 min, and hold until 10 min. Compounds were ionized by electrospray operating in positive ion mode with capillary voltage at 3.5 kV and S-lens RF level at 50. Spectra were acquired using a data-dependent MS/MS method with survey scans acquired at 35,000 resolution across *m*/*z* range of 70 to 700 and an AGC target of 3e6 and 200 ms maximum injection time. MS2 scans were acquired at 17,500 resolution with an AGC target of 3e6, a maximum injection time of 400 ms, isolation width of 0.8, and stepped normalized collision energies of 20, 40, and 80.

### Gas chromatography/MS

Samples for GC/MS were derivatized and analyzed by GC time-of-flight MS following the protocol described by Lisec et al. (2006). In short, the dried aliquot from the polar phase of metabolite extraction was derivatized using methoxyamine chloride and MSTFA. A mixture of fatty acid methyl esters (FAMES) was added to the samples for the calculation of retention indexes. Data processing was performed using the Xcalibur software, and metabolite annotation was performed based on the Golm Metabolome Database (Kopka et al. 2005).

### TQS micro tandem quadrupole MS

Samples for absolute cyclo(His-Pro) quantification were analyzed by liquid chromatography coupled with MS (LC–MS/MS) using a Waters Acquity UPLC interfaced with a Waters TQS micro tandem quadrupole mass spectrometer. Five microliters of sample were injected onto a Waters Acquity HSS-T3 UPLC column (2.1 × 100 mm). Compounds were separated using the following 13 min gradient: initial conditions were 100% mobile Phase A (10 mm perfluoroheotanoic acid, PFHA in water) and 0% mobile Phase B (acetonitrile), hold for 1 min at 100% A then ramp to 65% B at 8 min, ramp to 90% B at 8.01 min and hold until 9 min, return to 100% A at 9.01 min and hold until 13 min. The column temperature was 40 °C, and flow rate was 0.3 mL/min. Compounds were ionized by electrospray operating in positive ion mode with a capillary voltage of 1.0 kV, source temperature of 150 °C, desolvation temperature of 350 °C, cone gas flow at 40 L/h and desolvation gas flow at 800 L/h. Cyclo(His-Pro) concentration was determined using an external standard curve (8 different concentrations) and normalized to the sample tissue weight. Due to the unavailability of labeled cyclo(His-Pro), [^13^C^15^N]-labeled proline was used as an internal standard to correct for ion suppression.

### Stress experiment

Arabidopsis (*A. thaliana*) plants (ecotype Col-0) were grown in a long-day photoperiod (16 h day/8 h night), at 20/18 °C, under an irradiance of 150 *µ*mol/m^2^ s. At 4 wk, plants were randomized, and subjected to heat stress (37 °C), cold stress (4 °C), high-light stress (1,400 *μ*E), a combination of heat and high-light stress (37 °C and 1,400 *μ*E) and dark stress. Five independent plants were harvested separately at 0.5 and 6 h after stress onset constituting independent replicates.

### Salt-stress experiments for cyclo(His-Pro) analysis

Sterilized seeds of *A. thaliana* (Col-0) ecotype were placed in pretty square dishes and kept under dark conditions for 3 d. After breaking the vernalization, the plants were grown and cultivated in sterile conditions using a half-strength MS culture medium supplemented with 1% sucrose under long-day conditions (16 h of light and 8 h of dark) at ∼22 °C and a light intensity of 110 *μ*E for 12 d. To investigate the effects of salt (NaCl) on cyclo(His-Pro) accumulation, 12-d-old seedlings cultured on half-strength MS medium were transferred to MS plates supplemented with 100 mm NaCl or without NaCl as a control. Samples were collected at 0.5, 4, and 24 h after the treatment. Approximately 50 mg per biological replicate was flash frozen in liquid nitrogen and stored at −80 °C until analysis.

### Cyclo(His-Pro) supplementation experiment

Arabidopsis seedlings (ecotype Col-0) were grown in the liquid 0.5 MS medium with 1% sucrose in the 16 h/8 h day/night regime in the 6-well plate on an orbital shaker. The medium was exchanged after 10 d of growth, and on Day 12; plants were supplemented with either mock or 100 *μ*m cyclo(His-Pro). Treatment was applied at the beginning of the night, and seedlings were harvested at the 4 h time point.

### Isothermal shift experiments

Arabidopsis (Col-0) seedlings grown on the 0.5 MS medium with 1% sucrose in the 16 h/8 h day/night regime were harvested at 4 d after germination and immediately frozen in the liquid nitrogen. Frozen plant samples were pulverized using a mortar and pestle. Lysis buffer (50 mm Tris-HCl pH 7.5, 150 mm NaCl, 1.5 mm MgCl_2_, 1 mm phenylmethylsulphonyl fluoride, 1× Protease Inhibitor Cocktail, Sigma-Aldrich P9599, Steinheim, Germany) was added to the sample, 1 mL per 1 g of frozen plant material, and mixed until thawed. The resulting slurry was transferred to 2 mL centrifugal tubes and centrifuged for 10 min at 20,817 × *g* at 4 °C to remove cellular debris. Protein concentration in the obtained crude lysate was measured using the Bradford assay (Bradford 1976). Lysate was diluted with the lysis buffer to the final concentration of total protein of 1 mg/mL. One hundred microliter aliquots of the lysate were aliquoted to the PCR tubes. Cyclo(His-Pro), cyclo(Gly-Pro), and cyclo(Tyr-Asp) were added to the final concentration of 100 *μ*m. After 30 min incubation in room temperature, samples were heated in a PCR machine for 3 min at 53 °C followed by centrifugation for 10 min at 20,817 × *g* at 4 °C. Approximately 80 *μ*L of soluble fraction was gently transferred to a new tube, and the remaining protein pellets were discarded. Proteins from the soluble fraction were precipitated using 2.5× volume of cold acetone, overnight. After 10 min centrifugation at 20,817 × *g* at 4 °C, acetone was removed and protein pellets were washed with 0.5 mL of cold methanol, and left to dry in room temperature. Dried protein pellets were stored in −80 °C.

### Proteomics

Protein samples were processed as described in Thirumalaikumar et al. (2023). Briefly, a denaturation buffer (6 m urea, 2 m thiourea dissolved in 50 mm ammonium bicarbonate) was used to dissolve the pellets. A 60 min incubation at room temperature with DTT (100 *μ*m) served to reduce the proteins. These were subsequently alkylated by incubating with 300 *μ*m iodoacetamide (in darkness) for 60 min. Additional 10 min incubation with 100 *μ*m DTT served to quench residual iodoacetamide. Protein digestion using trypsin/Lys-C mixture (Mass Spec Grade, Promega) for 16 h was performed, according to the manufacturer's instruction, followed by desalting-step using C_18_ sep-pak column plates as described in Thirumalaikumar et al. (2023). The peptides were dried using speed vac and resuspended in a resuspension buffer (5% acetonitrile in 0.1% formic acid).

Approximately, 1 *μ*g of the peptides was injected for analysis. A nano-LC system (Dionex ultimate 3000) and an acclaim pepmap C_18_ column were used to separate the peptide mixtures, at a flow rate of 300 nL/min. The Solvent A/B gradient was as follows: being isocratic at 3% B for 15 min, linearly increasing to 45% B at 110 min, linearly increasing to 55% B at 115 min, keeping at 95% B from 130 to 131 min, shifting back to 3% B in 137.5 min and holding until 155 min. The peptide samples were sprayed using a nano-bore stainless-steel emitter (Fisher Scientific). Peptides were analyzed using an Orbitrap-Exploris-480 mass spectrometer. Data were collected using a data-dependent acquisition mode using a cycle time of 3 s. Standard mass spectrometer parameters were kept as described in Henneberg et al. (2023), briefly: positive ion voltage ∼2.3 kV, ion transfer tube temperature at 320 °C; full scan orbitrap resolution 120,000, scan range *m*/*z* 400 to 1,200, RF lens at 50%, maximum injection time mode was kept at Auto, AGC target was kept at standard, and normalized AGC target value was used; ddMS^2^ filters include monoisotopic peak determination for peptide, intensity threshold was kept at minimum intensity of 20,000, charge state 2 to 6, exclude isotopes; ddMS^2^ isolation window *m*/*z* 1.4, first mass (*m*/*z*) was set at 120, HCD normalized collision energies 30%, collision energy type was kept at normalized, orbitrap resolution 15,000, RF lens 50%, standard AGC target, auto maximum injection time. HeLa digests (Pierce, 88329) have been used to monitor the RT drift and mass accuracy of the LC before and after each experiment. Raw data were analyzed using the Proteome Discoverer (version 2.5, Thermo Fisher Scientific) following the manufacturer's instruction. PD Search was made using an *A. thaliana* protein database downloaded from UniPort. Common contaminants were compiled and added to the search. The search settings were as described in Wagner et al. (2025).

### Protein expression and purification

Recombinant GAPC1 and GAPDH were purified from an *Escherichia coli* expression strain engineered to express a His-tagged protein in the pET28b + vector. The constructs, including gene synthesis, were ordered from Bio Basic, Inc. The protein sequences of GAPC1 (P25858) and GAPDH (P04406) were retrieved from UniProt. The process began with a bacterial preculture in LB medium, which was then grown to the OD of ∼0.6. Protein expression was induced by adding 0.5 mm isopropyl thiogalactopyranoside (IPTG), followed by incubation overnight at 20 °C. Frozen bacterial cells were used for subsequent protein purification. The supernatant was loaded onto a 1 mL HisTrap (Cytiva) column connected to NGC Quest 10 (Bio-Rad), previously equilibrated with Buffer A [25 mm Tris-HCl pH 8.0, 300 mm NaCl, 5% (v/v) glycerol, 10 mm imidazole]. The column was washed with 10 mL of Buffer A, and the recombinant protein was eluted with a linear gradient of imidazole (10 to 300 mm, 50 mL). The fractions containing GAPC1 and GAPDH were collected and, subsequently, concentrated and desalted using ultra-centrifugal filters.

### Nano-differential scanning fluorimetry

Approximately 0.8 *μ*g of GAPC1 and GAPDH protein diluted using a reaction buffer (50 mm Tris-HCl pH 8.0) was used per capillary. Capillaries were loaded into Prometheus NT.48 (Nanotemper). Unfolding was detected during heating in a linear thermal ramp (2 °C/min, 20 to 90 °C). Temperature-dependent protein unfolding was determined from changes in tryptophan and tyrosine fluorescence at emission wavelengths of 350 and 330 nm. Melting temperatures were determined by detecting the maximum of the first derivative of the fluorescence ratios (F350 nm/F330 nm).

### Microscale thermophoresis

GAPC1 protein was labeled using the Monolith His-Tag Labeling Kit RED-tris-NTA 2nd Generation kit. Binding was performed in the 50 mm Tris-HCl pH 8.0 buffer supplemented with 1.6 mm NAD^+^, using 50 nm labeled protein and premium capillaries. Measurements were performed using the Monolith X instrument. Binding was analyzed from the spectral shift A670/A650 data using Monolith X software.

### GAPC1/GAPDH enzymatic assay

GAPC1 and GAPDH activities were monitored spectrophotometrically at 340 nm at 25 °C in an assay mixture containing 50 mm Tris-HCl pH 8.0, 1 mm Ga3P, 4 mm NAD^+^, and 10 mm Na_3_AsO_4_.

### NADP(H) and NADP(H)H measurements

NADP(H) measurements were performed, as described by Zhang et al. (2020).

### Salt-stress experiments for FW measurements

Arabidopsis Col-0 and *gapc1/2* seedlings were germinated on 0.5 MS medium. After 5 d, seedlings were transferred to fresh 0.5 MS plates, prepared with or without 125 mm NaCl. The plates were supplemented with DKPs at concentrations of 0.1, 1, and 10 *µ*m. FW was recorded 16 d after the transfer. In an independent experiment, sterilized Col-0 seeds were placed in square Petri dishes containing 0.5 MS medium supplemented with 100 mm NaCl, either with or without the addition of 100 *µ*m cyclo(His-Pro). FW was recorded after 12 d of germination.

### Statistical analyses

Significance was estimated using unpaired 2-tailed Student's *t*-test. A significance threshold of 0.05 (or 5%) was used to determine whether a result is statistically significant.

### Accession numbers

Sequence data from this article can be found in the UniProt data libraries under accession numbers GAPC1 (P25858) and GAPDH (P04406-1).

## Supplementary Material

kiaf174_Supplementary_Data

## Data Availability

Proteomics and GC/MS metabolomics data are available in Supplementary material. Raw proteomics chromatograms from the iTSA experiment were deposited to Mass Spectrometry Interactive Virtual Environment (Choi et al. 2020); MSV000097655, doi:10.25345/C52B8VQ68.

## Supplementary data

The following materials are available in the online version of this article.

**Supplementary Figure S1.** Identification and quantification of cyclo(His-Pro) in Arabidopsis samples.

**Supplementary Figure S2.** GAPC1 purification.

**Supplementary Figure S3.** Effect of dipeptides and amino acids on GAPC1 activity.

**Supplementary Data Set 1.** Proteomics datasets from the iTSA experiment.

**Supplementary Data Set 2.** Metabolite data from the GC/MS measurements.

**Supplementary Information (SI).** Synthesis and NMR validation of cyclo(Tyr-Asp).

## Dive Curated Terms

The following phenotypic, genotypic, and functional terms are of significance to the work described in this paper:

GAPC2 Gramene: AT1G13440
GAPC2 Araport: AT1G13440

TIR1 Gramene: AT3G62980
TIR1 Araport: AT3G62980

NADPH CHEBI: CHEBI:16474

NAD CHEBI: CHEBI:13389

NADP CHEBI: CHEBI:25523
