# Supplementary material for: Characterization of the cyclic dipeptide cyclo(His-Pro) in Arabidopsis
Source: Plant Physiol. 2025 May 3;198(1):kiaf174. doi: 10.1093/plphys/kiaf174 (PMC12089766; doi:10.1093/plphys/kiaf174)
Supplement: kiaf174_Supplementary_Data [file kiaf174_supplementary_data.zip › Supplement information.pdf]

Supplementary Data

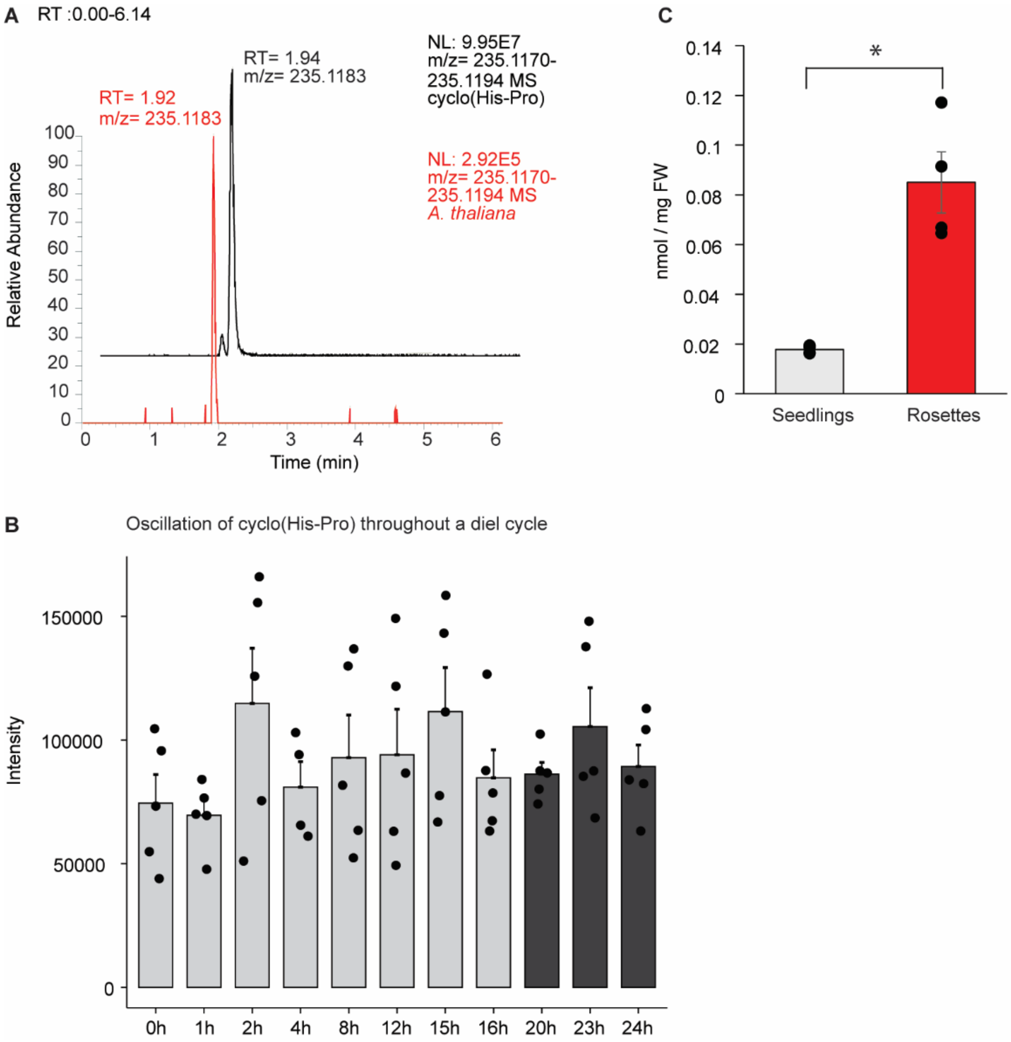

Supplemental Figure S1. Identification and quantification of cyclo(His-Pro) in Arabidopsis samples. A) Detection of cyclo(His-Pro) via C18 HPLC-HRMS. B) Oscillation of cyclo(His-Pro) levels in Arabidopsis thaliana Col-0 plants throughout a diel cycle (24 h), long day. Measurements derive from the C18 HPLC-HRMS method. Data are mean+/-SE of *n*=4-5, where replicates are independent plants. Figure was prepared using standard settings for a bar plot embedded in the SRplot web server (Tang et al., 2023). Data are from (Calderan-Rodrigues et al., 2021). C) Concentration of cyclo(His-Pro) in the 4-5 weeks old rosettes of Arabidopsis thaliana Col-0 plants grown in soil under standard, long-day conditions, and in two-weeks old Arabidopsis seedlings grown on 0.5 MS media supplemented with 1 % sucrose. Measured using TQS micro tandem quadrupole mass spectrometry and expressed as nmol / mg fresh weight (FW). Data are mean+/-SE of *n*=4 where replicates are independent plants (rosettes), or plates (seedlings). Significance was estimated using unpaired two-tailed student's *t*-test. A

significance threshold of 0.05 (or 5%) was used to determine if a result is statistically significant; indicated with Asterix. Figure was prepared using standard settings for a bar plot embedded in Excel.

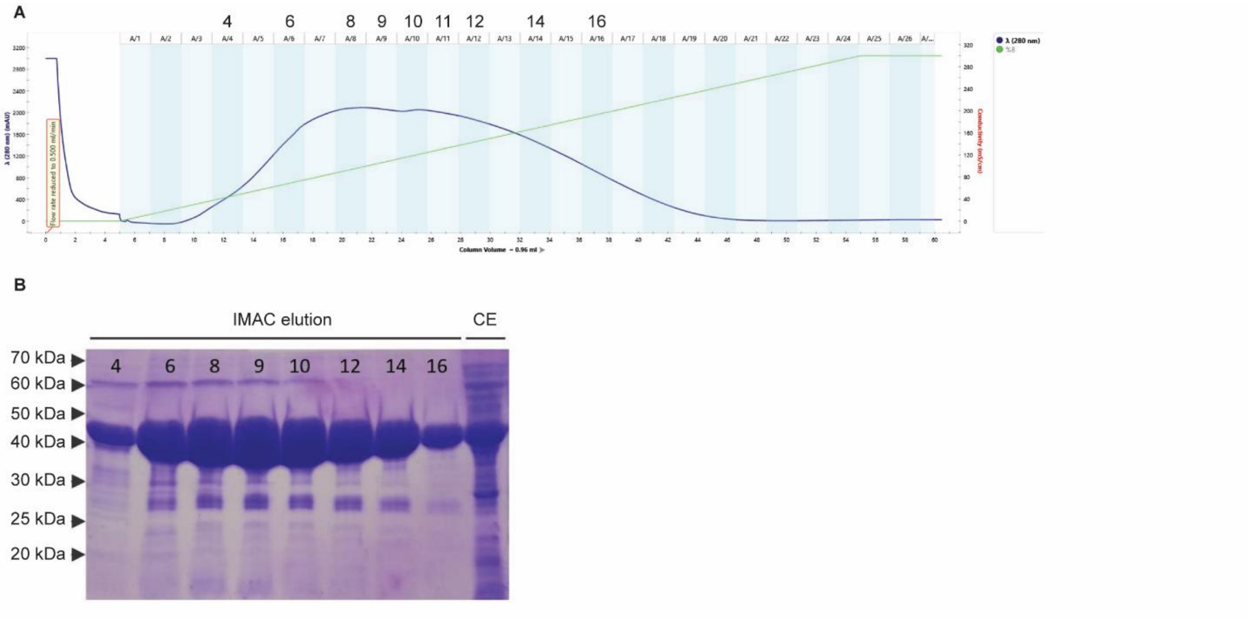

Supplemental Figure S2. GAPC1 purification. A) GAPC1 IMAC purification chromatogram B) and SDS-PAGE; numbered fractions in the gel corresponding to the chromatogram peak and the bacterial crude extract (CE) expressing the protein of interest with a monomer molecular mass of 45 kDa approximately.

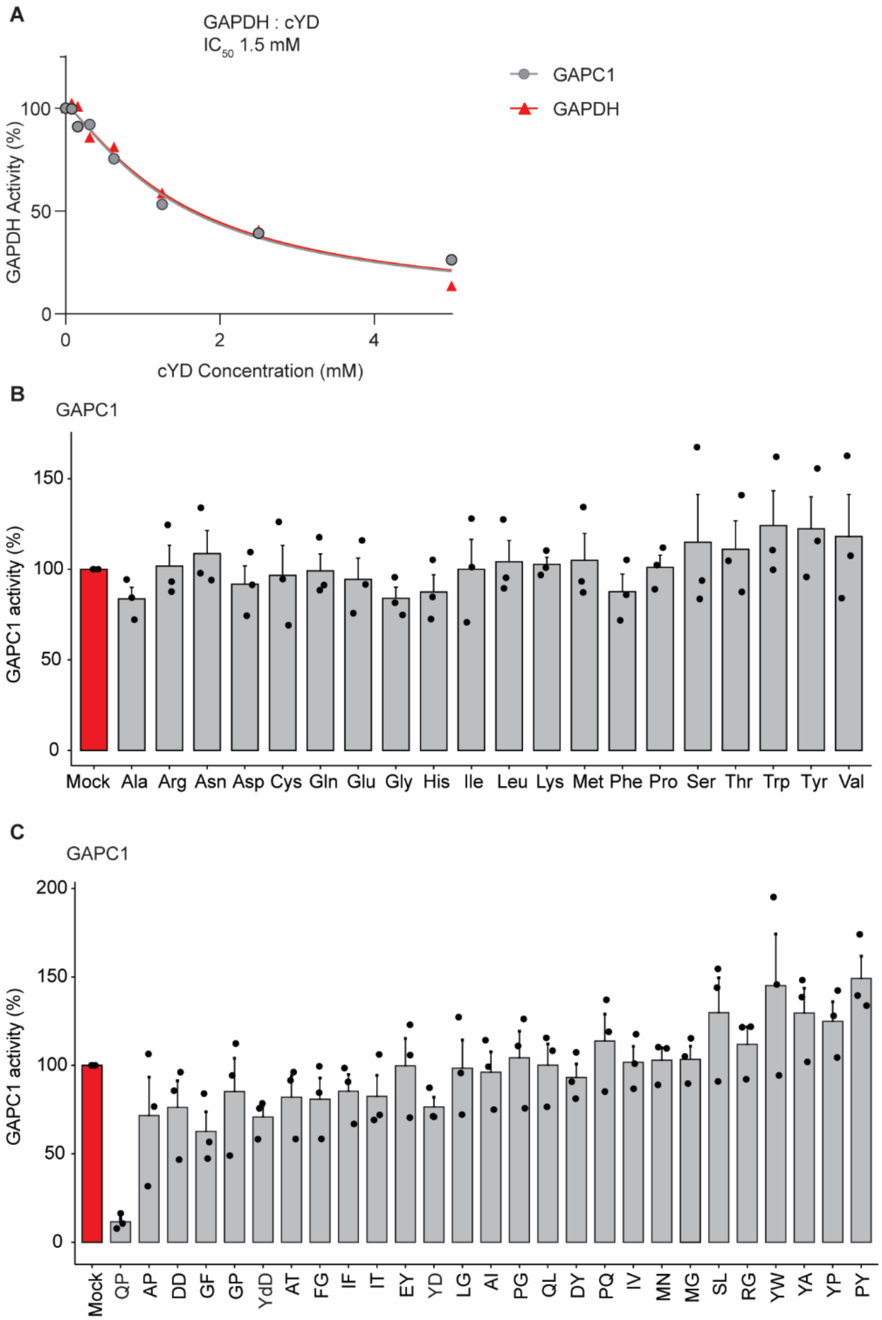

Supplemental Figure S3. Effect of dipeptides and amino acids on GAPC1 activity. A) GAPC1 and GAPDH activity measured in the presence of different cyclo(Tyr-Asp) concentrations. Given is an estimated IC_{50}. Data come for a single measurement. B) GAPC1 enzymatic activity in presence of different amino acids (10 mM final concentration, except for Tyrosine (2.5 mM). C) GAPC1 enzymatic activity in presence of different dipeptides at 10 mM final concentration, except for Asp-Tyr (3 mM), Glu-Tyr (2.5 mM), Met-Gly (1 mM), Pro-Tyr and Tyr-Ala (5 mM). B, C) Data are mean+/-SE of n=3, where replicates come from independent kinetic experiments. Figures were

prepared using standard settings for a bar plot embedded in the SRplot web server (Tang et al., 2023).

Supplemental Information (SI) Synthesis and NMR validation of cyclo(Tyr-Asp).

General synthetic procedures

All chemicals and reagents were purchased from Sigma-Aldrich unless noted otherwise. All oxygen and moisture-sensitive reactions were carried out under an argon atmosphere in flame-dried glassware. Solutions and solvents sensitive to moisture and oxygen were transferred via standard syringe and cannula techniques. All commercial reagents were purchased as reagent grade and, unless otherwise stated, were used without any further purification. Dichloromethane (DCM), and methanol (MeOH) used for chromatography and as a reagent or solvent were purchased from Fisher Scientific. Thin-layer chromatography (TLC) was performed using J. T. Baker Silica Gel IB2F plates. Flash chromatography was performed using Teledyne Isco CombiFlash systems and Teledyne Isco RediSep Rf silica columns. All deuterated solvents were purchased from Cambridge Isotopes. Nuclear Magnetic Resonance (NMR) spectra were recorded on Bruker INOVA 500 (500 MHz) spectrometers at Cornell University's NMR facility. All NMR data processing was done using MNOVA 14.2.1 (https://mestrelab.com/).

Synthesis of Cyclo(Tyr-Asp)

Compound **SI-1**

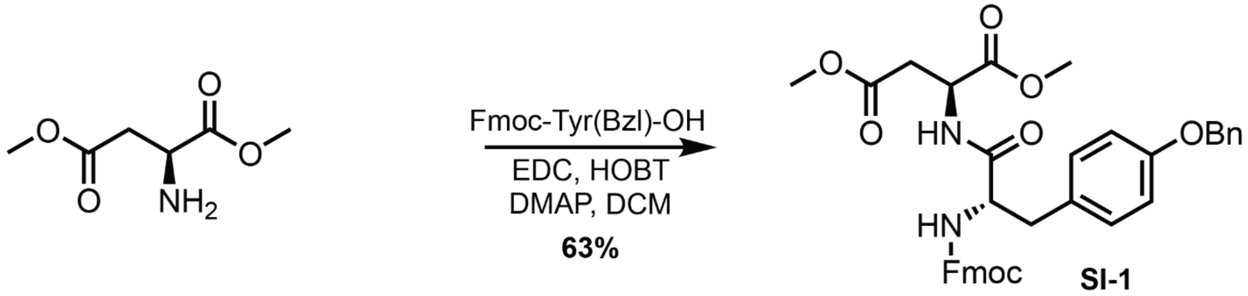

To a solution of Fmoc-*O*-benzyl-L-tyrosine (332 mg, 0.672 mmol, 1.5 eq) in DCM (4 mL) was added EDC·HCl (172 mg, 0.896 mmol, 2 eq), and hydroxybenzotriazole (121 mg, 0.896 mmol, 2.0 eq). The solution was stirred for 15 min at before adding DMAP (164 mg, 1.34 mmol, 3.0 eq), and **SI-1** (72 mg, 0.448 mmol, 1.0 eq). The mixture was stirred for 2 h, then concentrated *in vacuo*. The crude product was purified by flash column chromatography using a gradient of 0-100% MeOH in DCM, which afforded **SI-2** (72.2 mg, 63%).

Compound **SI-2**

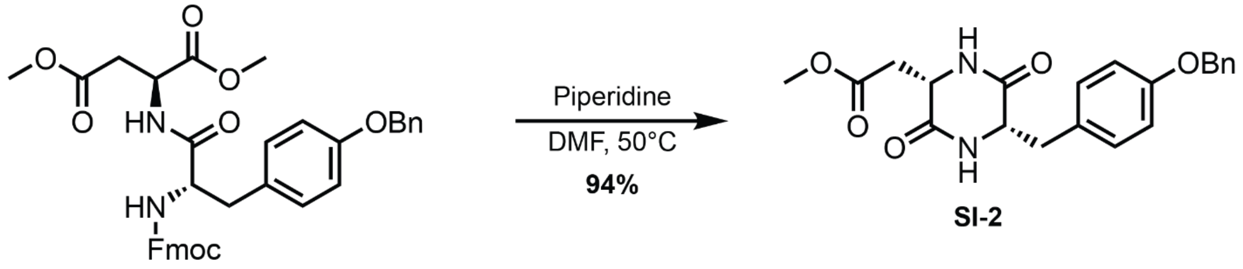

SI-1 **SI-2**

To a solution of **SI-1** (32.1 mg, 0.049 mmol, 1.0 eq) in DMF (4 mL) was added piperidine (500 µL, 5.06 mmol, 10 eq). The solution was heated to 50°C and stirred for 1 h, then concentrated *in vacuo.* The crude product was purified by flash column chromatography using a gradient of 0-100% MeOH in DCM, which afforded **SI-2** (20.6 mg, 94%).

Compound **SI-3**

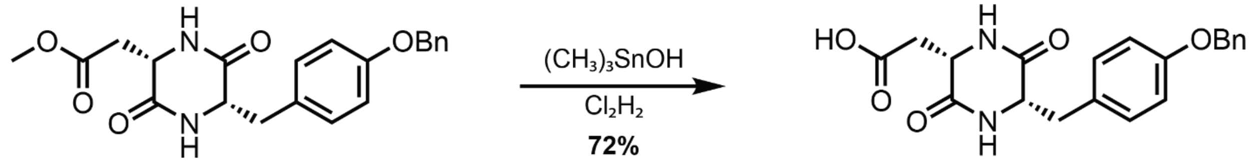

SI-2 **SI-3**

To a flame-dried round bottom, flask back-filled with argon, containing **SI-2** (10.0 mg, 0.027 mmol, 1.0 eq) was added a solution of trimethyltin hydroxide (50.0 mg, 0.27 mmol, 10.0 eq) in dichloroethane (5 mL). The reaction mixture was refluxed under an inert atmosphere overnight, then concentrated *in vacuo*. The crude product was purified by flash column chromatography using a gradient of 10-100% MeOH in DCM, which afforded **SI-3** (7.0 mg, 72%).

Cyclo(Tyr-Asp)

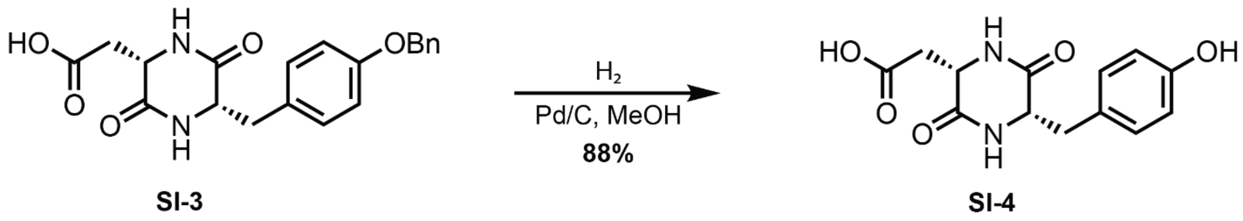

A suspension containing **SI-3** (5.0 mg, 0.013 mmol, 1.0 eq) Pd/C (14.0 mg, 10% w/w) and MeOH (2 mL) was sparged with argon for 5 minutes, then filled with H_2 and stirred vigorously for 90 min. The reaction mixture was filtered over celite, concentrated *in vacuo,* and purified by flash chromatography using a gradient of 10-100% MeOH in DCM, which afforded cyclo(Tyr-Asp) (3.2 mg, 88%).

1H NMR (500 MHz, methanol-d4): δ (ppm) 6.98 (d, J = 8.5 Hz, 2H), 6.69 (d, J = 8.5 Hz, 2H), 4.2 (td, J = 1.4 Hz, 4.9 Hz, 1H), 4.08 (ddd, J = 1.3 Hz, 2.6 Hz, 11.1 Hz, 1H), 3.11 (dd, 5.1 Hz, 13.9 Hz, 1H), 2.92 (dd, 4.5 Hz, 13.9 Hz, 1H), 2.51 (dd, 2.6 Hz, 16.7 Hz, 1H), 1.44 (dd, 11.1 Hz, 16.7 Hz, 1H).

13C NMR (500 MHz, methanol-d4): δ (ppm) 177.8, 169.7, 168.8, 160.3, 132.3, 125.6, 117.5, 57.5, 54.2, 42.1, 40.0

NMR spectra

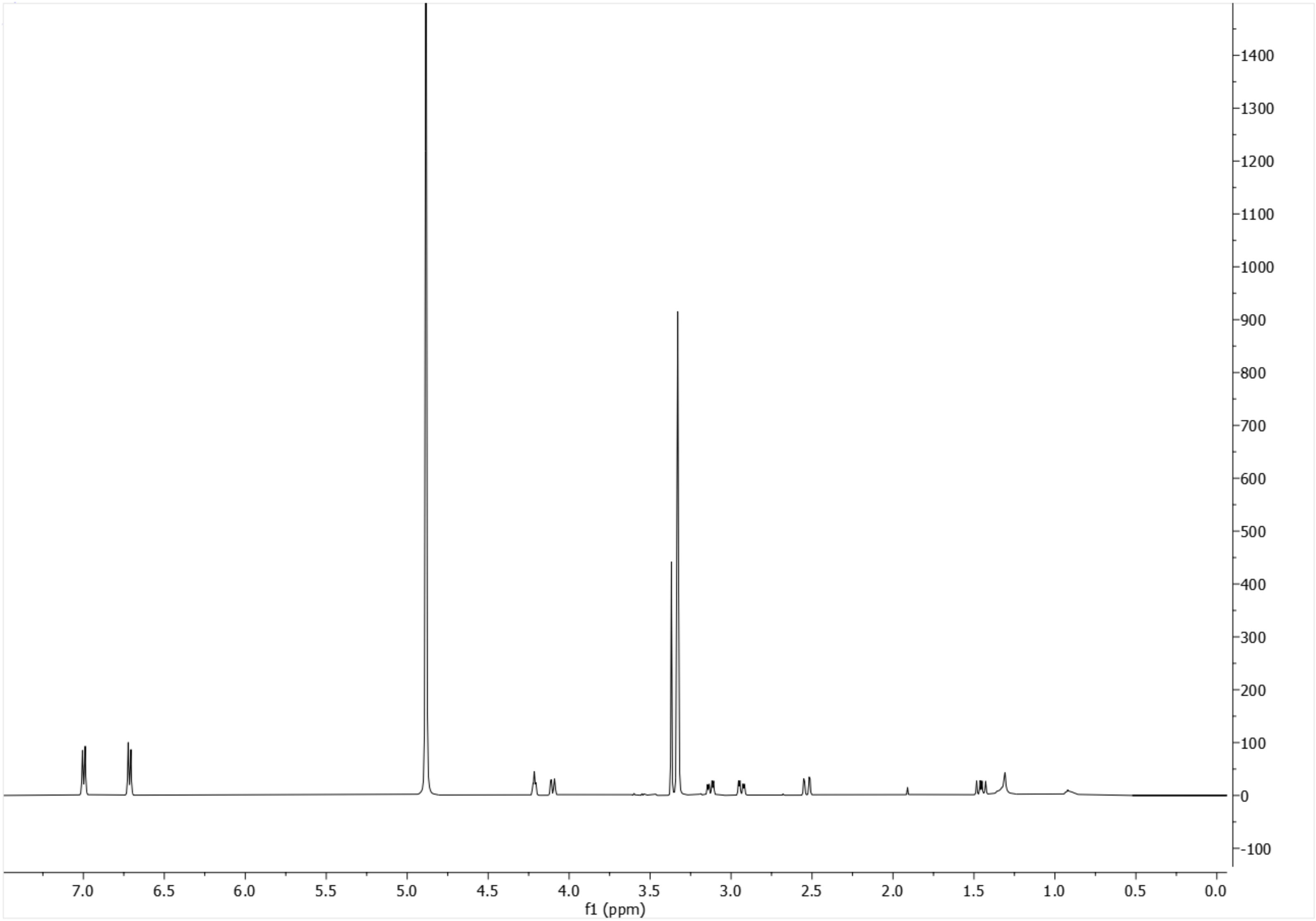

^{1}H NMR spectrum (500 MHz) of cyclo(Tyr-Asp) in methanol-d_4.

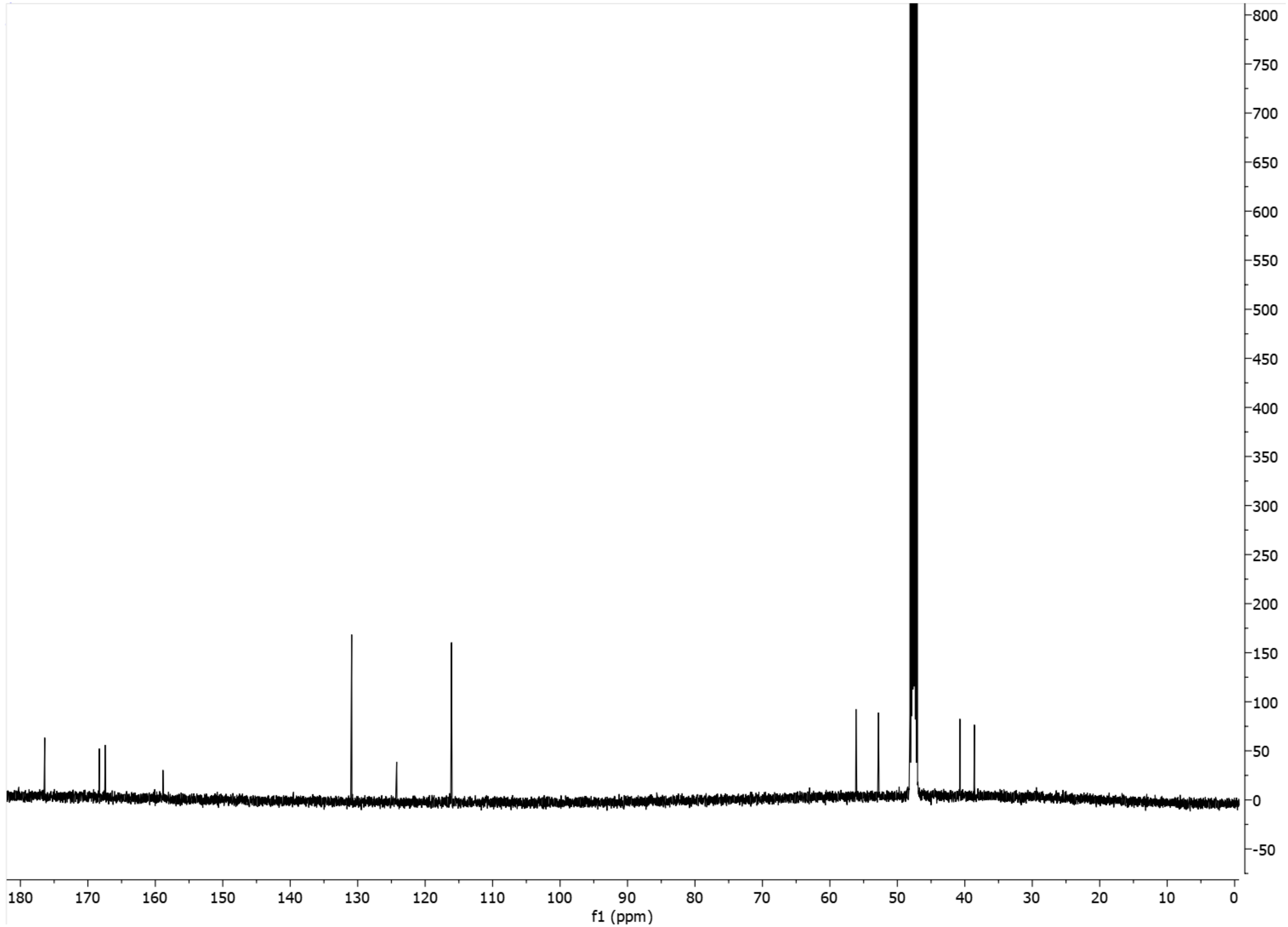

^{13}C NMR spectrum (500 MHz) of cyclo(Tyr-Asp) in methanol-d_4.

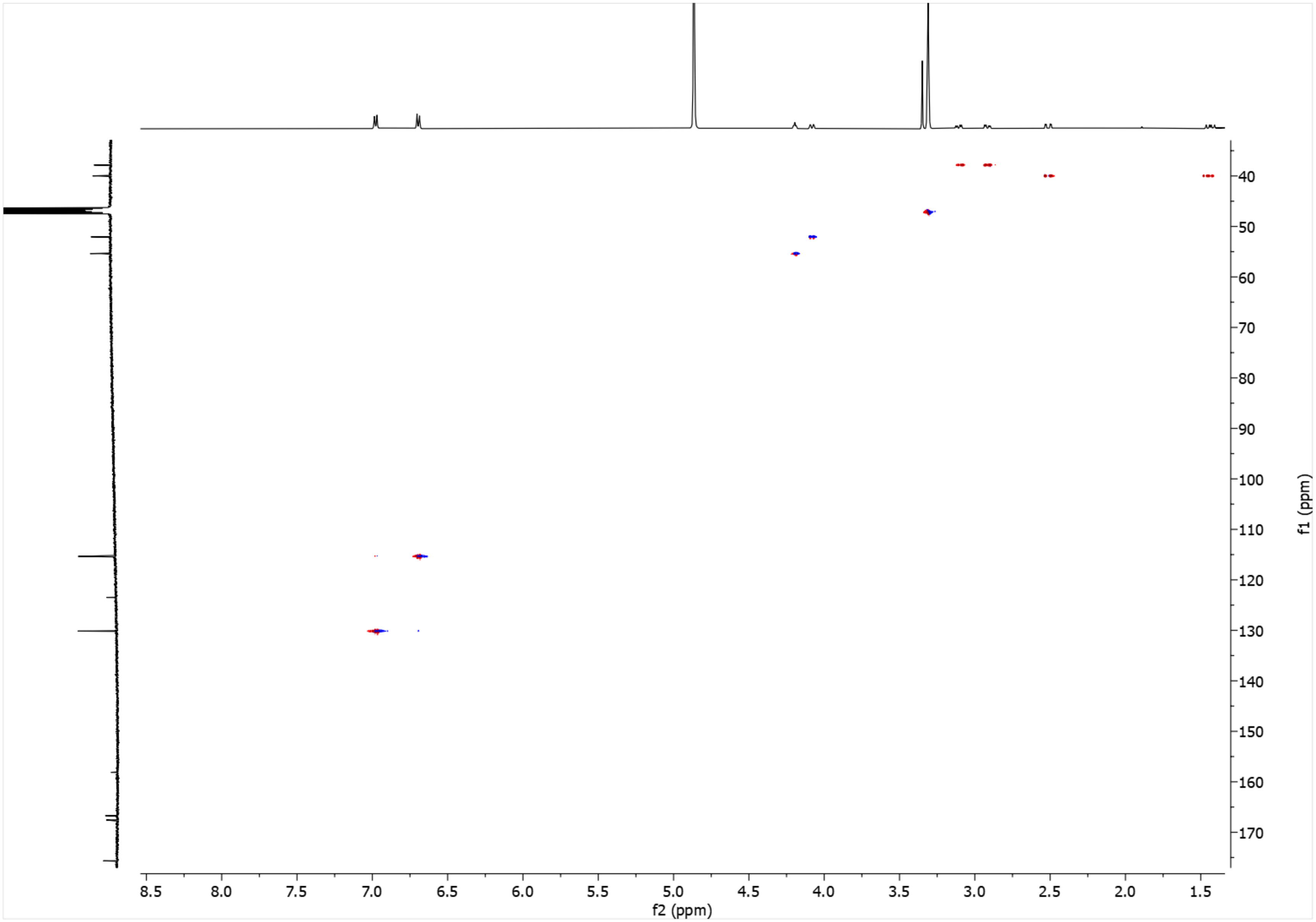

(^{1}H,^{13}C)-HSQC NMR spectrum (500 MHz) of cyclo(Tyr-Asp) in methanol-d_4.

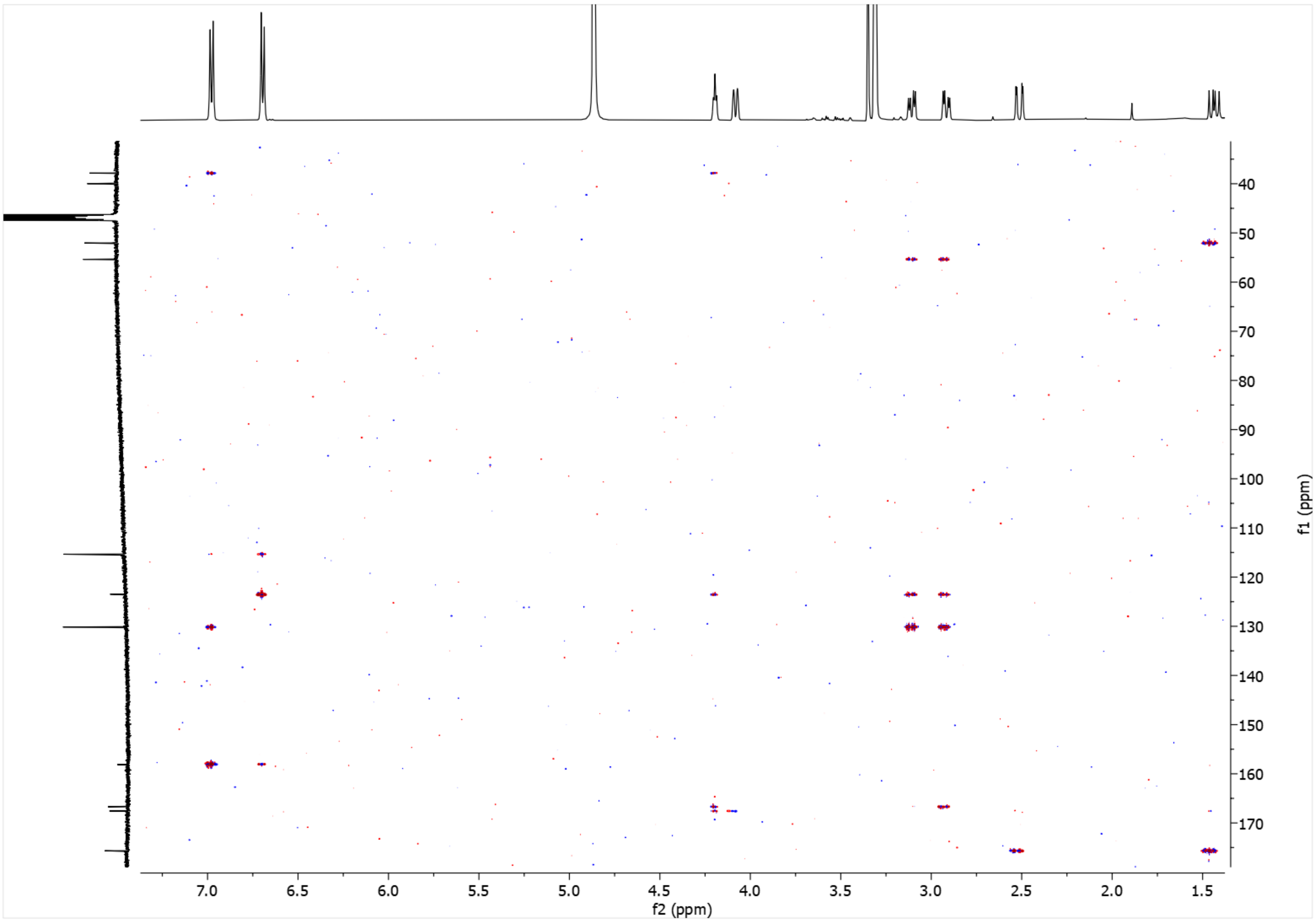

(^{1}H,^{13}C)-HMBC NMR spectrum (500 MHz) of cyclo(Tyr-Asp) in methanol-d_4.

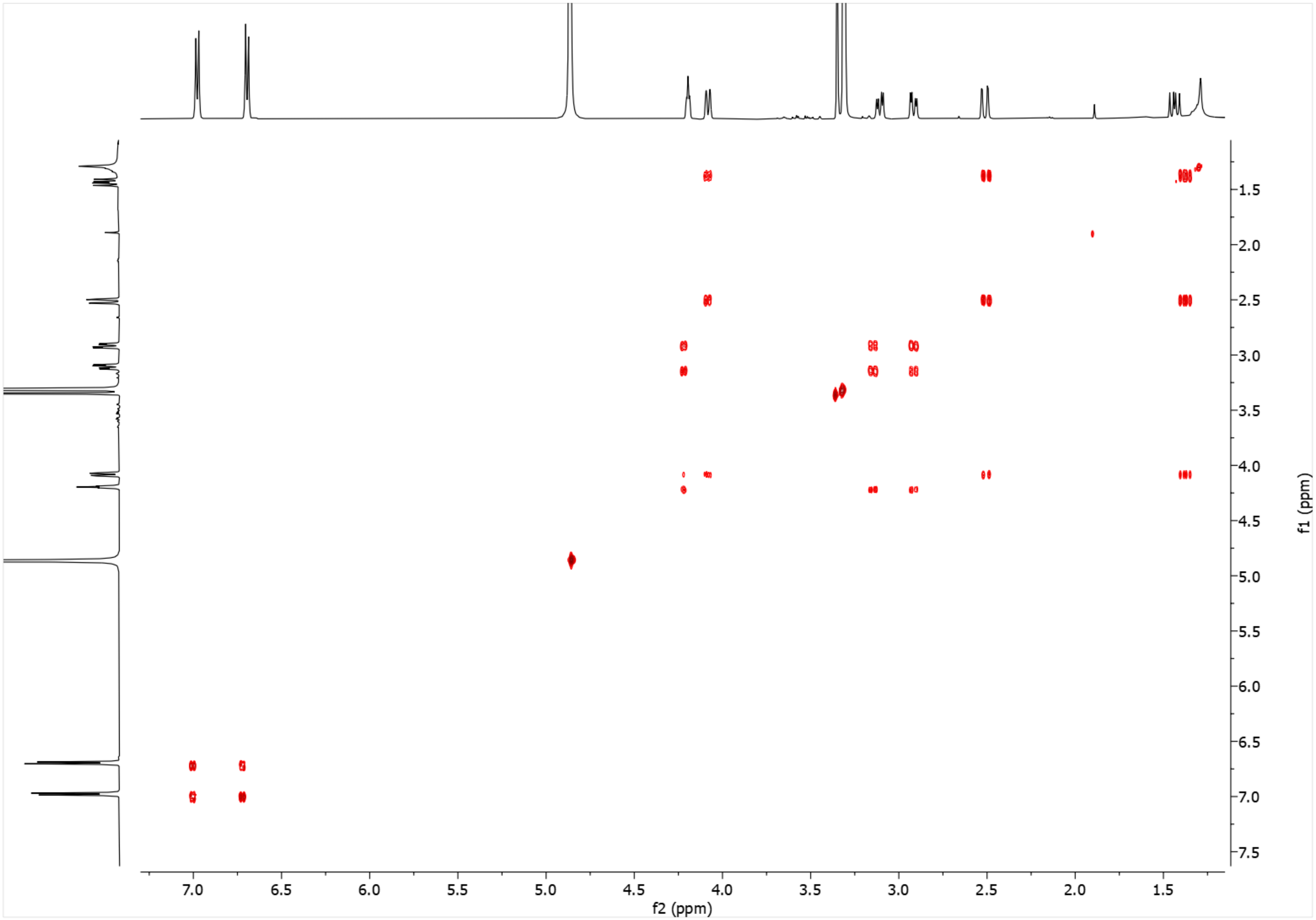

gCOSY NMR spectrum (500 MHz) of cyclo(Tyr-Asp) in methanol-d_4.
